# Effect of Salt Content Reduction on Food Processing Technology

**DOI:** 10.3390/foods10092237

**Published:** 2021-09-21

**Authors:** Jana Rysová, Zuzana Šmídová

**Affiliations:** Food Research Institute Prague, Radiová 7, 102 00 Prague, Czech Republic; jana.rysova@vupp.cz

**Keywords:** food reformulation, salt reduction, technology, salt replacement, spices, herbs

## Abstract

Higher salt intake is associated with the risk of cardiovascular and kidney diseases, hypertension and gastric cancer. Salt intake reduction represents an effective way to improve people’s health, either by the right choice of food or by a reduction of added salt. Salt substitutes are often used and also herb homogenates are treated by high pressure technology. Salt reduction significantly influences the shelf life, texture, pH, taste, and aroma of cheese. The composition of emulsifying salts or starter cultures must be modified to enact changes in microbial diversity, protease activity and the ripening process. The texture becomes softer and aroma atypical. In bakery products, a salt reduction of only 20–30% is acceptable. Water absorption, dough development, length and intensity of kneading and stability of dough are changed. Gluten development and its viscoelastic properties are affected. The salt reduction promotes yeast growth and CO_2_ production. Specific volume and crust colour intensity decreased, and the crumb porosity changed. In meat products, salt provides flavour, texture, and shelf life, and water activity increases. In this case, myofibrillar proteins’ solubility, water binding activity and colour intensity changes were found. The composition of curing nitrite salt mixtures and starter cultures must be modified.

## 1. Introduction

Salt is the most common ingredient for food flavoring—it is the only substance that gives a pure, salty taste. The perception of salty taste is influenced by customer age, sex, genetic equipment, weight, or the influence of smoking and medication. Increasing age is associated with a decreasing response of receptors to salty taste [1]. Obese individuals are more sensitive to this taste [2]. Research into brain function has shown that sodium releases the neurotransmitter dopamine in the brain, which is involved in the mechanism of motivation, emotions, and feelings of pleasure and reward; therefore, it is said that the use of salt is addictive.

Another point is the negative effect of sodium on human health. In industrialized and developing countries, the lifestyle and the way of eating have changed. The consumption of highly processed foods containing significant amounts of energy, (saturated) lipids, added sugars and salt has increased.

**Figure 1:**
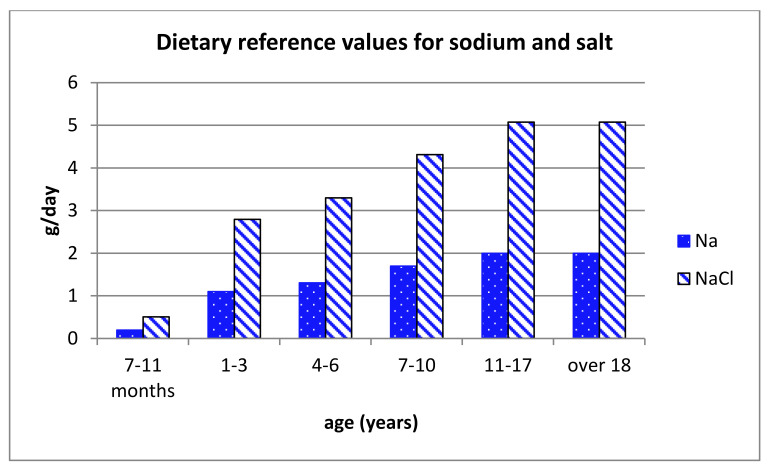
Sodium and salt dietary reference values for children and adults [3].

Sodium is an essential mineral for the body. The EFSA Panel on Nutrition, Novel Foods and Food allergens established a sodium intake of 2 g/day (corresponding to salt intake 5 g/day) as safe and adequate for the European adult population and corresponding lower doses for children [3] (Figure 1). 

**Figure 2:**
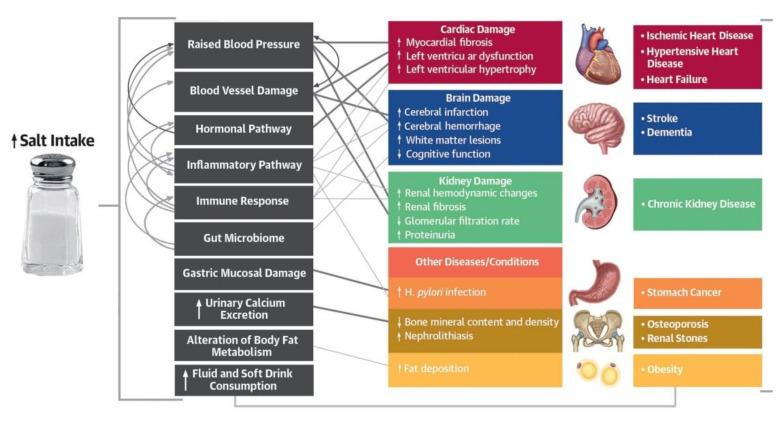
Effect of salt reduction on hypertension adapted from He et al. [13].

In practice, the long-term salt consumption higher than 5 g/day for an adult negatively affects the cardiovascular system and increases the risk of hypertension, heart diseases, and stroke [4,5,6]. High daily salt intake in some countries, 12–16 g/day [7], or 6.8–10.7 g/100 g based on urine excretion [8], causes kidney diseases [9,10], and indirectly affects the incidence of gastric cancer [11,12,13] (Figure 2).

**Figure 3:**
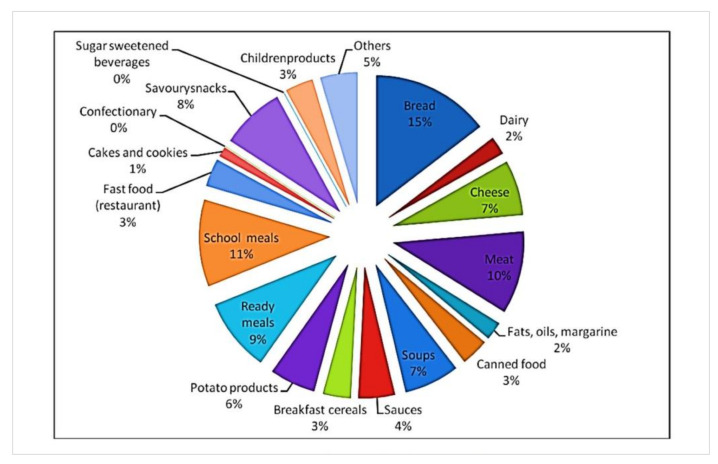
Food commodities on which reformulations aimed at salt content reduction are focused [14].

**Figure 4:**
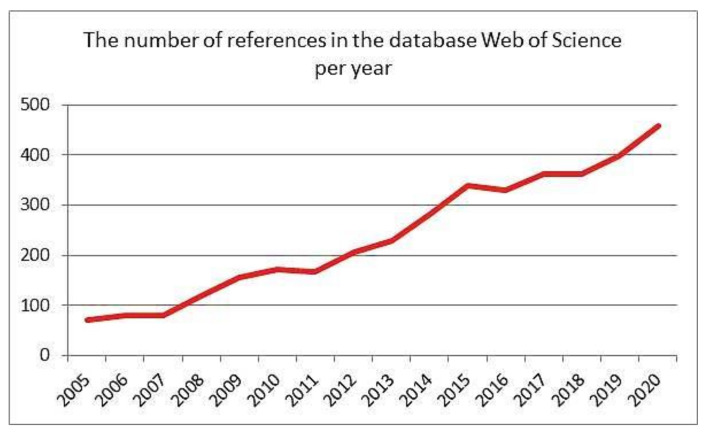
Web of science references research results on “salt reduction” and “food” key words [16].

The reformulation program supported by WHO includes among other nutrients restrictions also the reduction of daily salt intake [14] (Figure 3). Therefore, the WHO members have agreed to 30% reduction of salt consumption by 2025. This is the cheapest preventive measure to maintain human health [15]. In addition, the number of references in the Web of Science related to keywords “salt reduction” and “food” has increased during the years [16] (Figure 4).

The reformulation of food products is currently very topical; therefore, the purpose of this study was to bring comprehensive information about the possibility of reducing the salt content in food, and the effects of reducing the salt dose on bakery, meat and cheese products. The reformulated products with a lower salt content must be sensorily acceptable and must retain an adequate shelf-life. The information about changes in physicochemical properties, texture and sensory properties of individual foods as well as how these changes are reflected in fermentation processes and food shelf-life are summarized in this article. The novelty in this reformulation process is associated with the search for new technological possibilities of food processing, modified salt crystals preparation and also with the return to traditional natural raw materials in the form of processed herbs and extracts.

## 2. Sodium Occurrence in Foods and Possibilities of Its Reduction

In many foods of plant origin, salt is a minority element, but its content increases in food by up to several orders of magnitude by salting, seasoning or preservation. About 75% of the sodium ingested by food comes from sodium chloride or sodium glutamate, which are added during the production and preparation of food. In the report from 2007, the WHO [7] compares the sodium content of some raw, unprocessed, and subsequently processed foods, especially canned foods. As can be seen, industrial food processing increases the sodium content of selected foods. Some mineral waters also represent a significant source of sodium.

According to Regulation (EC) No 1924/2006 [17], the nutrition claims related to salt content are shown in Table 1. These values are not applicable for water, including mineral water.

There are three ways to achieve a salty taste while reducing sodium content:

(1) Influence taste receptors or signaling pathways with unsalted substances. So far, this direction is only theoretical.
(2) Find natural salt replacements that are non-toxic and metabolizable. In this way, partial or full substitution of NaCl with other salts like potassium chloride, magnesium chloride or calcium chloride [18] can be used. Furthermore, it is recommended to reduce salt intake gradually [19].
(3) Find non-toxic natural substances that enhance the taste of salt so intensively that it will be possible to reduce the salt dose. Amino acids and hydrolysates of proteins, nucleotides, spices, herbs and seaweed are used as salt enhancers [20,21].

The intensity of the salty taste can be reduced by modifying the salt crystals. Micronized salt with a purity of 99.5% is available on the market. It can be used in pastries and ready-to-eat meals. However, in meat and canned products, it does not serve optimally for salt content reduction because of its high moisture content in matrices [22,23,24]. The possibility of modifying the size, morphology and spatial structure of salt crystals was studied. Non-cubic crystals, hollow microspheres and agglomerated small salt crystals enhance the perception of salty taste. The salt surface increases and so does the interaction with taste receptors. The dimensions of salt particles are less than 100 μm, but it is also possible to prepare nanoparticles and the mixture of KCl and NaCl in a similar way. The structural modification of salt by spray drying or by electrohydrodynamic atomized drying results in particles with the size of 1.21 μm and 520 nm. These micro- and nanoparticles of salt dissolve rapidly in saliva and accelerate two to three times the perception of salty taste. Salt complexes with polysaccharides or proteins as carriers also reduce the NaCl content. These modified salts reduce the salt dose in bakery products, salted snacks, coatings and seasonings [25,26,27,28,29,30].

The overview of materials applicable as salt substitutes and salt enhancers are shown in Table 2.

### 2.1. Salt in Cheese, Bakery and Meat Products

Cheese is an essential part of the diet in the Western world. The salting of cheese has a direct effect on its sensory quality and technological properties. Therefore, salt reduction in cheese is difficult to put into practice [18]. Salt, along with a decrease in pH and other factors, affects the texture and the shelf life of cheese by blocking the growth of unwanted microflora. The salt contributes to the reduction of water activity, affects the charge of protein molecules, influences the solubility or, conversely, the aggregation of proteins or their hydration. It affects the activity of proteolytic enzymes and strongly influences the rheological properties of the raw material and the texture of the cheese itself by means of strengthening the surface of the cheese. Conversely, the exchange of sodium ions for calcium ions in casein softens the consistency of the cheese. Cheese with a higher pH has a softer consistency; hard cheese is more acidic. The salt causes an increase in the osmotic pressure between the curd grains and leads to the release of whey. Reduction of the salt content in cheese can be done without affecting the quality by slightly reducing the addition of salt, its partial replacement with potassium, ammonium, or magnesium chlorides, or modification of the technological process [18,31,32,33]. Although technologists have been concerned with salt substitution or its reduction for a long time, this issue has still been the subject of research and development.

Sodium plays an important role in the utilization of calcium from dairy products. Sodium has a calciuretic effect; the reason for this is a sodium–calcium exchange in the proximal part of the renal tubules where the calcium/sodium ratio is important. The higher this ratio, the lower the risk of renal calcium loss. For milk, this ratio is 2.7; for fresh cheese, it is 0.2; for some hard cheese, the ratio increases to 10. It was found that one gram of sodium above the recommended daily intake meant a loss of 20–40 mg of calcium from the bones [34]. Cheese usually contains 0.5–2% of salt, special cheese with mold inside and white cheese have a salt content of 3–7%. The salt penetrates the cheese mass by diffusion, so it also depends on the arrangement and size of the fat balls and the viscosity of the raw material during salting. Perfect and equal salting of the cheese mass is a matter of weeks to months.

Bread, pastry, and other bakery products contribute considerably to the high daily salt intake of industrially processed foods. The salt content of bakery products varies according to technology and taste habits in individual countries, ranging from 0.9 to 1.7%. The addition of salt to the pastry not only modifies the taste of the product but has primarily a number of technological consequences. The dose of salt in the bakery is usually expressed on the base of flour amount used and its quality is distinguished according to its granulation [35,36]. The salt of diametre of 0.25 to 0.50 mm is a fine ground salt applicable to the dough. Other salt types represent several groups according to their diameters: salt with diameter up to 1.25 mm, sprinkling salt up to 2 mm, followed by salt up to 2.8 mm and coarse salt up to 4 mm of diameter. The edible crystal salt comes from a modified crystallization process in vacuum, and it has good solubility with 1–2 mm granulation. It slowly gets wet and is used for dusting. The non-wetting edible salt may contain up to 1% of anti-caking agents. The salt is applied to the dough either as a loose raw material or in the form of various concentrated solutions up to a saturated solution.

One of the possibilities for maintaining the salty taste perception is the addition of coated salt crystals and their intentional irregular distribution in crumb. In this way, the salt content can be reduced up to 50%, and the perception of saltiness is maintained. However, there is a problem with local changes in the colour of the crumb. Larger, uncoated salt crystals can act in a similar manner. When laminating plunder dough, it is possible to intersperse the low-salt layers of the dough with more saline layers. Layers of less and saltier dough formed by lamination can also be prepared [37]. The effect of salt content reduction on the processing of plunder dough was manifested by less shrinkage of the dough during rolling. A lower dose of salt can be reached by incorporating encapsulated salt into the dough layer during lamination [38]. Salt agglomerates with waxy starch were prepared and irregularly distributed in salt-reduced bread. The use of salt agglomerates did not change the dough properties too much and the salty taste perception was increased. It was concluded that the 30% salt dose reduction is possible if the salt is used in the form of aggregates [39].

The addition of salt significantly affects the absorption of water in the flour and thus the development of the dough, the time required for its kneading and the intensity of kneading. During kneading, salt promotes the formation of the structure of gluten and its viscoelastic properties [40]. With the addition of salt, the dough is better mechanically processed and becomes softer and more flexible. The addition of salt to a flour dose is usually 1.5–2%; for low-quality flours, up to 2.5% of salt is needed to strengthen the dough. The reduced-salt dough is firmer at the beginning of kneading, but less stable and prone to over-kneading and the resulting dough is weak and sticky [41]. The ionic nature of sodium chloride leads to a broad interaction between water and the macromolecules of proteins and starch. These interactions then affect the moisture content and water activity in the bread. Water, on the other hand, is not sufficiently available for gluten due to the competition of salt, hydrogen, and sodium for binding sites on the molecule. There are electrostatic repulsive forces between the gluten molecules, which contribute to the formation of a fibrous protein network [42,43,44]. The network of gluten proteins and the salt content also contributes to the quality of puff and plunder pastry.

The bakery industry uses various substances as salt flavour enhancers. This group includes amino acids as well as trehalose, glutamate, and lactic acid salts. The taste is then complex. The roasted taste of malt or fermentation products such as soy sauce or bread yeast also contribute to the resulting taste [37].

Meat products contribute 20% of typical total salt intake. Therefore, there is an understandable effort to reduce the salt content in meat products, which, however, encounters limitations due to technology, consumer taste habits and product storage requirements. In meat products, sodium chloride is a multifunctional and indispensable component. In addition to enhancing the taste, it supports the shelf life of meat products, texture, colour, reduces water activity, and regulates the solubility of proteins. The meat contains a set of proteins that differ in solubility. The so-called sarcoplasmic proteins are well soluble in water and salt solutions. Myofibrillar proteins cannot be dissolved in water, but they are well soluble in salt solutions. A special role is played by stromal proteins, which are insoluble and form the basis of connective tissues. This division indicates how important the role of salt is in the processing of meat into meat products. Salt also has a role in proteolytic enzymes inhibition [32,45,46,47].

The salt dissociates into ions, which then perform their own function. As the ionic strength of the solution changes, the solubility of the myofibrillar proteins increases, these proteins then mediate the stabilization of emulsion in the meat mix and the adhesion of the meat particles. By heating and cooling, a gel is formed as the basis of the structure of the meat product. This function requires a salt content of around 2% [48]. The addition of salt alters the interactions between actin and myosin. These electrostatic interactions are based on negative and positive charges that can exert attractive or repulsive forces. The repulsive effect means more space between the protein chains. To use this process properly, a minimum dose of about 12 g NaCl per 1 kg of minced meat is required. Sodium ions also contribute to the formation of aroma, not only forming a salty taste, but also emphasizing other tastes. The salt content can only be reduced to a certain extent, then the functional properties deteriorate and the product spoils more easily. The product also has a less pleasant colour. The effect of short-term pre-salting on the colour of minced poultry pectoral muscle after cooking was also studied [46]. The meat was salted with 0–3% salt for 0–3 days and cooked. The yield of meat increased with salt content. At a 2% salt dose, myoglobin denatured rapidly, and higher salt doses also resulted in a higher nitrite content. If the addition of salt is 1%, a three-day meat loading is required for the colour development.

In meat production, the term water-binding activity (WBA) is very often used. This characteristic expresses the ability of the meat to bind its own and added water. The WBA of the meat depends mainly on the input quality of the meat given by the maturation process, on the pH and on the substances added to modify the technological properties of the raw meat material. The lowest WBA was found at a pH of around 5, and it is affected by both anions and cations contained in the salt. With increasing the addition of salt, the water-binding activity of the meat increases, but at about 5% salt dose, the trend reverses, and the binding activity decreases to the original values. The presence of salts of monovalent elements allows the immobilization of water in the meat; on the contrary, in the presence of divalent ions of calcium, magnesium, or iron, cross-links are formed between the protein chains, and the WBA decreases. In the context of the salt content reduction in meat products, an attempt to use meat before the onset of rigor mortis, which has a high water-binding activity, has emerged as an alternative method [49].

Salt is also used in the production of long-life meat products, including fermented products. During drying due to water loss, the salt concentration increases, and the water activity decreases. During fermentation, the pH decreases at the same time, so the salt content together with the low water activity and pH ensures the shelf life of the product. A part of this process also is affected by the starter cultures selected for the given conditions. Pure sodium chloride is used in meat production only in cooked meat products, bacon, and white sausage specialties. For most meat products, industrially produced nitrite salt mixtures are used, which have replaced the traditionally used long-term treatment of the meat with nitrate additions. The nitrite mixtures ensure not only the desired colour of the meat product but also promote meat product shelf-life and safety, because nitrite blocks the growth of *Clostridium botulinum* and acts as an antioxidant [50]. The salt mixtures usually contain 0.5–0.6% or, in a special embodiment 0.8–0.9% of sodium or potassium nitrite. Nitrites can only be added to meat as a part of a salting mixture. This is a safety precaution because sodium nitrite is toxic at higher doses. Nitrite in meat is reduced to nitric oxide, which reacts with myoglobin molecules to form nitroxymyoglobin (nitrosomyoglobin)—a typical pink dye of meat products. According to Regulation No 1333/2008 [51], the maximum permitted dose of nitrite in a meat product is 150 mg/kg expressed as sodium nitrite. For selected types of traditional meat products, other residual nitrite content of 100–175 mg/kg is allowed.

In the meat product preparations, salt in the amount of 2–3% is added to increase the myofibrillar proteins’ solubility. The form of the addition depends on the specific production technology. It can be added into the mixed minced meat product or by means of loading into brine for days to weeks, by spraying brine into the meat through needle systems or blood vessels, or by dry salting.

In practice, salt is a cheap carrier of other technologically important substances, such as spice extracts. It is put into the mixed minced meat in a cutter or mixer at the end of mixing. This manufacturing operation should take place in the cold so as not to unnecessarily release fat and disrupt the product appearance in the cutlet. Salt is also incorporated into larger pieces of meat during massaging (tumbling), where mechanical activation of proteins occurs, a part of the muscle proteins is released into solution and a viscous adhesive layer is formed on the surface of the meat pieces.

Other sodium-containing additives used in meat products are sodium ascorbate, sodium isoascorbate (reducing agent for products with salt mixtures), sodium lactate and sodium acetate (for longer shelf-life, reduced water activity, additions up to 1–2%), polyphosphates (adjust the WBA of meat and reduce production losses, bind calcium ions; they are not added to long-life products) and caseinates.

### 2.2. Spices and Herbs as Salt Substitutes

Spices and herbs have been used by humans for thousands of years. Recently, we have seen a negative attitude towards highly processed foods and a return to natural raw materials among some consumers. Therefore, the use of herbs is also relevant in connection with salt. Salt has served as a preservative for centuries, and herbs contain a number of antimicrobial agents. At the same time, experiments are being performed with herbs as a substitute for synthetic preservatives, as well as experiments with the use of the antioxidant potential of some herbs and spices. The salty taste is found in salt-loving herbs (halophytes) and seaweed. Among the commonly used herbs and spices, there are a number of palatable plants that could partially replace the salty taste of the dish. For example, Ghawi et al. [52] prepared low-salt tomato soup with added herbs and spices. The incorporation of herbs and spices enhanced the salty taste to the level of the control sample without salt restriction. An attempt to numerically express the ability of an herb to replace salt based on sensory analysis was made by Kohri et al. [53]. It was found that the addition of 0.35% of herb extracts enhanced the salty taste perception of an 0.4% salt model solution by 1.13–1.22 times. It is possible to prepare a homogeneous mass by mixing the fresh plants, treat this mass with high pressure and then use it as a semi-finished product in various dishes [54,55]. The herbs homogenate preparations represent a proven method of fresh herbs processing without heating or freezing and a method when biologically active substances are preserved [56].

The overview of plants applicable as seasonings and contemporary as salt replacements was compiled by Taladrid et al. [57]. Garlic is considered a good alternative to salt because of its strong taste, general acceptability, and health effects. Plants from *Lamiaceae* family are traditionally used for centuries. Mint, rosemary, oregano, basil, or thyme belong to this family, too. For this plant group, a high content of flavonoids, terpenes and phenolic acids is typical. Mixtures of herbs have also been used for many years. They are typical for a specific region or a certain type of food. Their effect is complementary and synergistic. Saffron is a rare spice derived from the flower of *Crocus sativus* with expected health effects. Hot spices, for example chili pepper, black pepper, ginger, and wasabi, enhance the perception of salty taste and reduce the craving for salty food. Grape-derived seasonings help to maintain the shelf-life of products, enrich them with fiber and phenolic substances and protect products from oxidation. In the Mediterranean basin, people have been used some wild edible species as foods or medicinal plants for ages. Many of these plants grow in saline soil, are accustomed to conditions near the sea and contain salt. These plants are picked up and used as medicines, spices, or vegetables. Petropoulos et al. [58] found many chemical components contributing to the health promotion or protection, better food shelf-life or improvement of the food taste and aroma. Herbs like *Suaeda fruticosa*, plants of *Salicornia* genus, *Beta vulgaris*, subsp. *maritima, Salsola soda*, *Portulaca oleracea, Capparis spinosa* and many others belong to this group of plants. In gourmet cuisine, *Sarcocornia perennis* subsp. *perennis*, *S. perennis* subsp. *alpini, Salicornia ramosissima* and *Arthrocnemum macro-stachyum* are consumed. These plants are characterized by high proteins and n-3 polyunsaturated fatty acids contents and show an important content of mineral compounds and tocopherols, and a high radical scavenging activity [59].

Other herbs commonly used in the kitchen are also able to replace the decreased intensity of the salty taste with their taste and aroma. Here can be included, for example, lovage (*Levisticum officinale*), curry plant (*Helichrysum italicum),* garlic mustard (*Alliaria petiolata*), coriander leaves (*Coriandrum sativum),* arugula (*Eruca sativa*), summer savory (*Satureja hortensis)*, fennel leaves (*Foeniculum vulgare*), rosemary (*Rosmarinus officinalis*), lemongrass (*Cymbopogon citratus*), nori and kelp algae (genus *Porphyra),* onion species (*Allium cepa*), garlic (*Allium sativum* and *Allium ursinum*), chive (*Allium schoenoprasum*), basil (*Occimum basilicum*), parsley (*Petroselinum crispum*) and many other plants. The addition of herbs allows for a 50% salt reduction with the same intensity of saltiness perception [60].

The halophytes of *Salicornia genus* (glasswort) belonging to family *Amaranthaceae* are known as a sea vegetable. The dried Salicornia powder can be used as a salt replacement. *Salicornia brachiata* contains a mix of biologically active compounds, especially natural antioxidants, flavonoids, and essential fatty acids [61,62]. Biologically active compound found in *Salicornia europaea* were investigated [63]. The content of alkaloids, tannins and flavonoid pigments were determined. Methanolic extract of *Salicornia fruticosa* was successfully used to inhibit growth of bacteria, yeasts, and moulds. The 1.5% extract or *Salicornia* juice was the most effective and extended the shelf life in samples of refrigerated minced beef [64]. A hydrate prepared by mixing glasswort, carrageenan, carboxymethyl-cellulose, soy proteins, and caseinate was added to the low-sodium Frankfurter sausages. The colour of new products changed, but cooking loss decreased, and the hardness and juiciness were improved [65]. It was proposed that the salt content in bread could be partially replaced by glasswort with its natural salty taste and high minerals content [66]. Plants of *Salsola* genus (saltwort) typically grow in the arid and semi-arid areas. Some *Salsola* species are used in cosmetics or for medical purposes because of their antibacterial and healing properties. The saltwort powder was successfully used as a salt alternative in the amount of 0–3% to the pork patties. Texture parameters of control samples were better than of samples with saltwort addition, but the sensory parameters were evaluated as better in salt-reduced samples [67].

Fresh seablite (*Suaeda maritima*) is used in Thai foods as a vegetable or as a dried product. The antibacterial activity of the seablite extract against a wide spectrum of Gram-negative and Gram-positive bacteria and multiple antibiotic-resistant strains were observed. The fungicidal and antioxidant activity were also manifested [68].

The original spices or herbs can be replaced by oleoresins encapsulated with carrier because of protection of aromatic substances from high temperatures during food preparation. The mixtures of *Allium schoenoprasum, Anethum graveolens, Capsicum frutescens, Mentha pulegium* or a mixture of *Allium schoenoprasum, Satureja montana, Capsicum annuum, and Origanum vulgare* were extracted. The obtained oleoresins were encapsulated into inulin or maltodextrin and dried. This standardized product can serve as a salt substitute in foods [69].

## 3. Salt Content Reduction and Its Consequences

### 3.1. Salt Reduction in Cheese Products

Reduction of the salt content or replacement of sodium by potassium is possible in the production of cheese only to a certain extent, since then the texture and aroma change occur and the cheese loses its typical character. This especially applies to cheese with higher salt content.

There are three types of cheese salting:

1. Grain salting, where dry salt is added to the ground or sliced curd at the end of processing before molding (Cheddar). The advantage is the short diffusion distance; even salinity is achieved in 20 min.
2. Dry salting carried out by repeated salt spreading on the surface of the cheese. In this way, the proteins on the surface of the cheese are withdrawn and diffusion is reduced.
3. Salt bath salting, which is used with most cheese, where the salt solution contains 18–22% of salt. The pH of the solution is 5.2 for hard and 4.8–5.0 for soft cheese; the salting process takes place at a temperature of 10–14 °C. In addition, 0.1–0.2% of calcium is added to the salt bath. When using a salt bath, contamination by yeast and fungi can occur, and even a specific microflora tolerating acidic pH, and higher salt levels can develop. In contrast, lactic acid bacteria are inhibited by salt [70].

To maintain the shelf-life, suitable combinations of a salt alternative and antimicrobial agents for the elimination of listeria, salmonella and coliform microorganisms were sought in the model conditions. Lactoperoxidase, laurylarginate, and fermentates were effective, but salt substitute were found to reduce the effect of fermentates and laurylarginate against Salmonella and *E. coli* [71]. Modifications of the technological process are also possible, which means mainly the inclusion of heat treatment in the production process in order to prevent the growth of undesirable microorganisms.

Quite often, the salt content reduction or its replacement is tested on Cheddar-type cheese, where the optimal salt content is related to the moisture content. An S/M ratio in the range of 4.7–5.7 is considered the most appropriate. Further reduction of the salt dose will cause a soft to greasy cheese texture and an aftertaste. The interaction of fat and salt in the production of Cheddar cheese was also monitored. The salt dose was reduced by half (0.9%), and the fat content ranged from 16–33%. Decreased salt content resulted in more broken casein and decreased cheese firmness. Reducing the fat content had the opposite effect as reducing the salt content [72]. The same authors [73] reduced the fat and salt content of Cheddar by 30–50% and observed changes during 270 days of cheese ripening. Reducing the salt content from 1.9 to 1.2% increased the moisture, water/lactic acid ratio, and water activity and reduced the residual lactose content. A decrease in fat content also caused moisture to rise and the protein/lactic acid ratio to decrease. The pH decreased in the control cheese and increased in the cheese with half the dose of salt or fat; for the other samples, it is constant. After 270 days, control cheese had lower lactic acid content, lower pH, and lower free amino acids content than cheese with salt and fat reduction.

As the salt content decreases, the amount of whey released from the curd decreases. Lu and Mahon [74] performed a model experiment with a raw material for Cheddar-type cheese. Salting was performed for 5 and 10 min and with different doses of salt (20–30 g/kg) and different numbers of applications. Salt substitution with 33% of KCl was also studied. Decreasing the salt content increased whey retention and slightly lowered the pH. Faster addition of salt did not increase whey release, but partial replacement of salt by the addition of KCl was effective. The addition of calcium ions also increased the syneresis of the curd, which then had a higher dry matter content. The amount of calcium bound to paracasein was proportional to the concentration of calcium in the solution. Murtaza et al. [75] reached similar conclusions. They reduced the salt content in Cheddar-type cheese from buffalo milk in the range of 2.5–0.5%. Reducing the salt dose lowered the pH and increased the humidity and water activity. Cheese hardness, firmness and friability decreased, but proteolysis increased. The concentration of volatile flavours increased, but the resulting aroma was evaluated negatively. The authors stated that salt could be reduced, but the formation of aftertaste should be regulated by a suitable starter culture.

A partial replacement of sodium in salt by potassium in Cheddar cheese with a total salt content of 1.7% was performed by McMahon et al. [76]. After salting, the pH decreased rapidly and a typical change in the microflora was found. The content of lactococci decreased, while the number of NSLAB (non-starter lactic acid bacteria) increased. As the salt content decreased, the zone for NSLAB vegetation widened. Potassium salts and other flavourings and bitter taste masking agents were used to reduce the salt content in Cheddar. Saloni K, a plant protein hydrolysate and adenosine-5′-monophosphate (AMP), provided the most salty and least bitter taste. As high as 75% salt replacement was achieved [77]. Ganesan et al. [78] tested the properties of Cheddar and mozzarella cheeses with a reduced salt dose from 0.7–1.8%, i.e., the sodium content was reduced by 25–36%. Nevertheless, cheeses with a higher salt content were rated better. Additional partial flavours were found in the sensory profile, which is slightly reflected in the limited salted samples, while in the saltier samples they are already covered by the dominant taste. The consumer recognized the reduction in salt content, but a 30% reduction in salt dose was feasible. The salt content can be reduced depending on the water content, the fat content, the pH and the content of probiotic microorganisms. It also depends on the ripening temperature of the cheese and storage.

Another suitable cheese for reducing salt is cottage cheese, where a 35% reduction in salt addition has been achieved. Cottage cheese with reduced salt content and additions of KCl or MgCl_2_ were prepared. The growth of probiotic bacteria *Lactobacillus acidophilus*, *Bifidobacterium lactis* and the growth of *Listeria monocytogenes* after intentional inoculation were monitored. *Listeria monocytogenes* were able to grow under these conditions, depending on the temperature (4–12 °C) and the addition of probiotics. Probiotics blocked the growth of the pathogen even in samples with a lower salt content [79].

Replacing the salt with 30–50% magnesium or calcium chloride proves to be the easiest in terms of the composition of the cheese, its texture and aroma. Another possibility is the addition of flavour enhancers; for example, with the addition of yeast autolysate to cottage cheese [80].

A white cheese was prepared from ultrafiltered cow’s milk with a salt content of 1.0, 2.5 and 4.0% by Soltani et al. [81]. The salt content affected the composition, proteolytic activity, number of lactic acid bacteria (LAB) and sensory quality of the cheese during 90 days of ripening. The number of LAB decreased with increasing salt dose. Cheeses with 1.0 and 2.5% of salt were sensory acceptable, as the aroma of the cheeses depends on the degree of proteolysis. Kamleh et al. [82] studied the effect of 30% KCl salt replacement on the quality of fresh and ripe Akawi cheese. Reducing the salt content affected the cohesiveness, chewability and hardness of the cheese. The content of microorganisms increased with the ripening time of the cheese, but no differences between the samples were noted. The effect of salt on the bitterness, friability and hardness of the cheese was noted, while the length and ripeness of the cheese had a greater effect on the colour and aroma. The effect of KCl salt replacement in Akawi cheese during storage was also discussed by Gandhi et al. [83]. The saline solution contained 10% salt; 7.5% salt; 7.5% mixture of NaCl and KCl (1:1); 5% NaCl or 5% of NaCl and KCl mixture (1:1). After 30 days, more probiotic bacteria survived in the lower salt cheese. The calcium content decreased slightly in all samples. The lower salt cheese extract improved the growth of cultured normal human colon cell and the release of some essential amino acids such as phenylalanine, tryptophan, valine, and leucine. The reduction in salt content did not affect the activity of potential bioactive peptides in the cheese extract.

Reducing the salt content of mozzarella cheese, often in combination with reducing the fat content, yields conflicting results. Arboatti et al. [84] prepared mozzarella cheese with a reduction in salt content of 35% and 60%. The main difference between the cheeses was in the intensity of the salty taste, but other parameters (humidity, water activity, transition temperature, sensory parameters) were not significant. No significant differences were also found in pH, casein degradation, and bitter taste intensity. Other authors [85,86], on the contrary, tried to reduce the salt and fat content at the same time and the results were not very satisfactory. The salt content of the cheese was reduced from 1.8% to 1.0% and the fat content from 23% to 11%. The cheese was then stored for 35 days at 4 °C. The reduction in fat content significantly worsened the sensory properties, the cheese gained greater strength and was rubberier, and the aroma of the cheese was weakened. Reducing the salt content had less significant consequences. Attempts were also made to prepare mozzarella with partial replacement of the salt with potassium chloride and the use of potassium citrate and diphosphate instead of sodium salts. The use of 31% salt replacement by KCl and 2% of emulsifying salts proved to be optimal. The control cheese had a sodium content of 905 mg/100 g and a sample of newly prepared cheese only 260 mg/100 g.

Salt replacement by potassium chloride was also tested in soft cheese. NaCl/KCl mixtures were prepared in a ratio of 3:1, 1:1 and 1:3. Salt replacement did not affect the basic composition and microflora of freshly formed cheese, but there was a greater decrease in pH. After 17 days of ripening, the moisture content of cheese with KCl decreased significantly; on the other hand, the hardness and friability of the cheese increased. Sensory evaluation and technological properties suggested a real possibility of a maximum of 25–50% KCl as a salt replacement [87]. The effect of the reduced salt content on the properties of soft-ripened cheese with mold on the surface was tested by Dugat-Bony et al. [88,89]. The control cheese with 1.8% salt and the sample with 1.3% salt were monitored for 27 days. Changes in the composition of microorganisms in favor of gram-negative bacteria and thus changes in proteolytic activity, aroma, and production of biogenic amines were recorded. In the sample with lower salt content, a higher content of Pseudomonas fragi was found, which is responsible for food spoilage. In the latter study [80], the NaCl content was either reduced (20%) or partially substituted with potassium chloride in soft Camembert-type and semi-hard Reblochon-type cheeses. In soft cheeses, the salt content influenced proteolysis at three weeks of ripening. In semi-hard cheeses, reduced salt content increased spoilage bacteria growth (Pseudomonas). Luzzi et al. [90] assessed the microbiological composition of reformulated Edam cheese. Reduction of sodium content by simple sodium reduction or by salt substitution with salt substitute containing potassium showed no influence on its microbial diversity.

The influence of different salt content on the activity of starter cultures is important. In the production of semi-hard cheese, the cheese samples had a salt content between 0.3–3.4%. The cheese matured for 12 weeks. The reduction of the salt content resulted in an increase of the LAB content at the beginning of maturation, especially in the unsalted sample. In the next phase of maturation, the effect of salt no longer manifested itself significantly. There was a negative correlation between salt content and proteolytic activity (PepX activity). Since the starter cultures affect the texture and aroma of the cheese, a new starter culture suitable to produce less salty cheese should be bred.

In the hard cheese of the Grana type, Tidona et al. [91] reduced the sodium content using brines with different amounts of potassium chloride. In cheeses salted with KCl-brine a lower weight loss was observed. The use of KCl-brine with the highest potassium concentration of 53.8% halved the sodium content in comparison with the control. After the ripening, cheeses had no defects and the partial replacement of sodium with potassium did not affect the chemical composition, microbiological composition, or ripening process, respectively. Moatsou et al. [92] assessed the influence of added denatured whey proteins together with NaCl/KCl-brine salting on sheep cheese. After determination of moisture on non-fat substances, they found it was similar in full-fat and reduced-fat cheeses (approx. 57%) and the indices of proteolysis were not influenced by treatments.

One of the possibilities to reduce the sodium content is the addition of flavour enhancers, e.g., arginine, yeast extract and oregano extract [93]. In the sodium-reduced Brazilian prato cheese, the use of flavour enhancers modified its sensory characteristics: yeast and oregano extracts increased the saltiness of the cheese, and arginine influenced mainly its characteristics. Furthermore, yeast extract addition showed a positive effect on the flavour because it reduced bitter taste caused by the addition of KCl.

The effect of probiotic strains of microorganisms may be useful for improving the shelf life of cheese. For example, the microbiological quality of low-emulsifying salt cheese analogues with added spores of the probiotic *Bacillus coagulans* was monitored during 60 days of storage. The result was a decrease in humidity, water activity and pH and a change in the proteolytic index of the control sample and the sample with probiotics. The proportion of surviving probiotics decreases with storage. After 60 days of storage, an increase of 20–67% in the total number of coliform bacteria and fungi was observed, but, on the contrary, a decrease in samples with probiotics was achieved. Samples containing probiotics were more resistant to spoilage [94].

### 3.2. Salt Reduction in Bakery Product

#### 3.2.1. Effect of Salt on Yeast Activity

Sodium chloride regulates to some extent the process of fermentation. Salt is usually not added to the fermentation pre-stages because its addition increases the osmotic pressure and inhibits yeast vegetation, suppressing CO_2_ production. Therefore, the salt should not come into direct contact with yeast, which liquefies and inactivates. At the same time, the addition of salt allows the leavening rate of the dough to be regulated as needed. The over-leavened dough would be too sour and the structure would be very poor. Because the vast majority of water is bound to macromolecules, salt is present in higher concentrations in the remaining free water. This concentration then acts on the yeast together with the osmotic pressure, the content of available sugars and the activity of the enzymes. The effect of salt addition on the fermentation rate, when CO_2_ production is used as a measure of yeast activity, shows that at 1%, 2% or 4% flour salt the fermentation is decreased by 6%, 20% or 70%, respectively [41].

Changes in dough are related to the salt dose [36]. The dough without added salt is flowable and the product has a dull taste. A dose of salt of 0.5% supports the growth of yeast, 1–1.5% of salt hardens the proteins, prolongs the kneading time, and reduces the softness and flexibility of the dough. The product has a better shape. At 2% salt, the fermentation process and the activity of enzymes slow down; at 2.5% salt, the dough rises slowly and is firm. A higher salt addition makes the product oversalted.

#### 3.2.2. Effect of Salt on Texture and Final Quality of Bakery Products

All parameters of the dough and fermentation are reflected in the quality of the final product. Baked product from dough with a reduced salt content results in changes in the texture of the crust and its colour, specific volume and sensory and aroma profiles [95]. During the leavening of less salty dough, the yeasts consume free sugars and therefore, fewer reducing sugars are available for Maillard reactions. There is a small number of large pores in the crumb and the crumb is unbalanced due to the weak network created by the gluten. Reduced salt content leads to lower gas volume in the bread dough [96]. The specific volume may ultimately be low if the weakened network does not maintain the expansion of steam and CO_2_ during baking. When baking puff pastry, the volume of the bread is also dependent on the salt dose [42]. The final quality of the bread is to some extent determined by the control of the water activity. Breads with a salt content of 0.3 and 0.6% versus 1.2% in the standard differed only little in volume, water content and baking losses. The changes were manifested during storage of bread on the staling process due to the starch retrogradation. The salt-reduced bread was dry and crumbly, standard bread with 1.2% of salt retained an acceptable texture. Salt affected water activity, dehydrated the cells and inhibited the metabolism of microorganisms. It also acted as a preservative against microbial spoilage and fungi [34,44]. The reduction of the salt dose in the pastry had to be compensated by other antimicrobial measures.

The intensity of the salty taste also depends on the rate of salt released from the food matrix during chewing in the mouth and the rate of salt transported to the taste receptors, i.e., also on the texture and viscosity of the food. This was demonstrated when breads with the same dose of salt but different leavening times were baked. They differed in pore size and thus in the rate of salt released into the mouth during chewing. The intensity of the salty taste was then perceived differently; bread with larger pores was considered saltier [97]. The salty taste and the taste of other food ingredients can enhance or attenuate each other. The result of these processes is the overall taste profile of the product [37]. For example, a 0.17% salt promoted the sweet taste of the amino acids glycine, alanine and serine. The salt also attenuated the bitter taste. Without the addition of salt, bread was rated as yeasty-tasted and sour.

Technologically, bread without salt or with a very low salt content can be baked but its sensory quality is poor. Salt reduction needs the modification of technologies, the other composition of raw materials and even the use of sourdough. However, any changes must also be cost-effective and consumer-acceptable.

#### 3.2.3. Reduction of Salt in Bakery

In sensory tests, the salt content of 1% was identified as ideal for the bread loaves. From the ideal content (395 mg Na/100 g bread) a 32% reduction was possible (to 267 mg Na/100 g of bread) [98]. If the salt dose is to be reduced, even partially, it must be reduced gradually. As an example, a 35% salt content reduction (from 1.7% to 1.1%) was achieved during three years without consumer detection [99]. In case of a rapid decrease, it is possible to use salt substitutes or flavour enhancers; in industrial production it is mainly potassium chloride. A relatively new technique is the formation of differently salty layers of dough or the use of larger salt crystals [100].

##### Simple Salt Dosage Reduction and Use of Salt Substitutes

Due to the technology used, it is not easy to reduce the salt content. The effect of sea salt with a sodium content of 57–64%, less than in conventional salt, on bread technology was observed by Miller and Jeong [101]. The effect of the selected salt on the dough tension, kneading time, carbon dioxide production, bread volume and crust quality was not observed. This bread was sensory rated slightly worse than the control bread. Kovac and Knific [102] monitored the preferences of preschool children for breads with a reduced salt dose by 30–50%. Bread with a salt reduction by 30% was accepted without problems; however, bread with half the salt content was evaluated as less acceptable. There is also the possibility of heterogenous NaCl distribution which led to increased salt perception due to the taste contrast in wheat breads [103].

To reduce the salt content in bread, a combination of salt substitutes—potassium and calcium salts, mostly in the form of phosphates and glutamates, is used. Lithium chloride is toxic and ammonium and lithium iodides and gluconates are not sensory acceptable. Potassium salts are the most effective substitutes, but a foreign taste was also reported. With a planned 25% reduction in salt consumption, the intensity of the aftertaste is not very noticeable [37,104]. La Croixa et al. [105] looked for a percentage reduction in salt dose. A 10% difference in the salt dose remained unrecognized; a 20 and 30% difference in saltiness was already detectable but did not affect the consumer’s acceptability of the product. The effect on the rheological properties, extensibility and stickiness of the dough was observed, but bread with 37.5% KCl instead of salt had similar properties as the standard sample [106].

The reduction of the salt dose from 2% to 1% in durum wheat bread was tried by Spina et al. [107]. Potassium chloride and yeast extract were chosen as partial substitutes. Bread packaged in a modified atmosphere was stored at 25 °C for 120 days. There were no significant changes in the texture and moisture of the bread. The content of 5-hydroxymethylfurfural did not depend on the content of salt or salt substitutes. The number of microorganisms during storage is growing, especially in less salty samples. The samples differed initially in salt content, flexibility and moisture. During storage, the moisture content of all breads decreased significantly. A bread with salt substitution mixtures of equal parts of CaCl_2_/CaCO_3_ was prepared by Basset et al. [108]. A total of 1.8% salts (based on flour) were added to the bread. A higher calcium content caused less elasticity and extensibility of the dough. The upper crust was stiffer and the lower softer than the standard; the colour of the crumb and crust was lighter. Potassium citrate is a salt substitute in commercial bread that has been used successfully in Spain [104]. Inorganic salts—KCl, MgCl_2_, CaCl_2_, Mg_2_SO_4_ and Na_2_SO_4_—were tested on three samples of commercial flour as substitutes for sodium chloride in the range of 0, 25, 50 and 100%. Due to the addition of these salts, the mixing properties and stickiness of the dough were modified.

On the farinograph, a small effect was found with potassium chloride. On the contrary, sodium sulphate showed the maximum strengthening and compacting effect, and magnesium and calcium chlorides weakened the dough. With the addition of salts, the viscosity of the suspension increased when heated in all flours, mostly due to sodium sulfate. Magnesium, calcium and potassium chlorides improved the stability of the dough. At 25% salt replacement the viscosity of the suspension increased; however, at a higher dose of salt replacement, the viscosity decreased [109].

Another possibility offers the use of natural low-sodium sea salt Saltwell^®^. Breads containing 0.15–1.70% Saltwell^®^ (based on flour) were prepared. Using different salt types, no significant differences in bread volume, yield, or sensory perception over the storage time (over 90 days) were observed, but texture differences were found. The lowest content of 5-hydroxymethylfurfural was found in bread with the lowest level of Saltwell^®^. What’s more, its content even decreased during storage [110].

The effect of salt content in the range of 0–8.4% based on flour and simultaneous fat reduction by 40% on the properties of puff pastry were also tested. The effect of salt reduction on quality is significant. The dough with a full dose of fat with increasing salt content is less firm. Regardless of the fat content, pastries with a higher salt dose are of better quality as far as volume, texture and colour are concerned. It is still possible to reduce the salt dose by a third and the fat by 40% [42].

Li et al. [111] proved a method for salt content reduction in white bread by means of dietary fiber fortification using low-molecular-weight water-extractable arabinoxylans (LMW WEAX). This enables a 20% salt reduction without a negative impact on salt perception. The addition of pre-hydrated LMW WEAX allowed a 30% salt reduction resulting in low-salt high-fiber breads.

##### The Application of Sourdough

The trends to reduce the salt content also brought an effort to use of sourdough from wheat or rye as a means of extending the shelf-life and improving the character of the dough and sensory properties of bread with a lower salt content [42,112,113,114]. Fermentation of the wheat flour substrate with a strain of *Lactobacillus plantarum* gave a preparation which enhanced the taste and enabled a partial salt replacement in bread in the range of 20–50%. No difference in glutamate and free amino acid contents was found between the new bread and the standard one, but some acetic and lactic acid derivatives affecting the aroma of the bread were present in the new bread with the addition of fermented material [115]. The use of a sourdough preparation obtained by the fermentation with *Lactobacillus brevis* and a protease in an amount of 21% was tested. While the use of KCl did not affect the chemical composition and biological activity of bread, the use of sourdough increased the content of gamma-aminobutyric acid and peptides smaller than 3 kDa with the potential to act as ACE (angiotensin-converting enzyme) inhibitors and with higher antioxidant activity [116].

Dried sourdough was also added as a flavour enhancer in rolls. The salt content was 0–2.5% and dried sourdough was added in an amount of 0–10% based on the flour. Salt content reduction shortened the kneading and leavening time. The addition of sourdough prolonged the mixing and leavening time and at higher concentrations reduced the diameter of the rolls. Salt reduction up to 45% was achieved at a sourdough content of 5% while maintaining technological properties [117]. Zhao et al. [118] prepared sourdough fermented by selected strains of *Lactobacillus reuteri* and monitored the accumulation of gamma-aminobutyric acid (GABA). The assessors in the sensory analysis were able to recognize a 6% addition of sourdough. They were also able to distinguish the use of sourdough with GABA accumulation or sourdough with glutamate accumulation. Such breads containing 1% of salt were comparable to breads with 1.5% of salt. In addition to the salty taste, a sour and umami taste were identified in the taste profile. Jimenez-Maromoto et al. [119] compared bread with salt and bread with salt replaced by a fermented soy preparation. The addition of soy preparation changed the colour of the crumb and crust to dark and also affected the volume of the bread. The 25% salt replacement proved to be the best.

An experiment was performed to reduce the salt content in bread to 0–1.5% based on flour and its effect on the quality of bread prepared by different technologies was tested. Three technological methods of bread production were selected—the use of yeast, the classic method using bread sourdough and the procedure with the addition of starter culture. The salt content reduction did not affect the yield of the bread but had a significant effect on the volume, porosity and strength of the crumb. Differences were also found between the individual technological processes. The salt content reduction was mostly pronounced in bread with starter culture and bread with leavening ensured by the addition of yeast. The smallest differences were found for sourdough bread [120].

### 3.3. Salt Reduction in Meat Products

Salt affects not only the taste, but the overall quality of the ready prepared meat products or semi-finished products. Therefore, it is necessary not only to replace the missing strong taste, but also to solve the problem of maintaining an acceptable texture and shelf life of the product. The salt-reduced products should not significantly change from the original product [121]. For this purpose, it is possible to use another salty tasting substance (potassium chloride, lactate, or calcium ascorbate) and flavour enhancers (see Section 3.3.2.).

#### 3.3.1. Salt Replacement in Meat Products

The minimum level of salt in meat products was determined using sensory analysis and technological properties evaluation by Delgado-Pando et al. [122]. They found out that the salt reduction in bacon by 34% and in ham by 19% was feasible without the need for salt replacement.

Usually, the partial replacement of the salt with potassium chloride is used, and the taste is then adjusted with one of the enhancers. The disadvantage of salt substitution with KCl is the bitter taste; other salts are accompanied by a metal-like aftertaste. These undesirable flavours can be partially masked. The salt substitute can be further composed of calcium and magnesium salts, potassium citrate or lactate, glucose, nitrite, adenosine monophosphate to mask the bitter taste, and glycine. Phosphates and hydrocolloids may also be present in the salting mixture to maintain the WBA of the meat. Fungal products also appear as functional components [40]. The water-binding activity of model gels from myofibrillar proteins and the effect of polyphosphates and KCl were studied by Carkcioglu et al. [123]. With 50% salt replacement by KCl and tripolyphosphates, KCl reduced the flexibility of the resulting gel and, conversely, tripolyphosphates improved the functional properties of the gel. In another model experiment, poultry myofibrillar proteins were mixed with salt to a concentration of 0.6 mmol/l and 1.0 mol/l with the simultaneous addition of 5 mmol/l of histidine. Gel formation after heating was monitored. The solubility and gelling ability of the proteins increased, and the mobility of water in the gel decreased. Histidine is therefore considered as a flavouring in products with a lower total salt content [124]. The effects of KCl, MgCl_2,_ and CaCl_2_ were also studied in the gel from myofibrillar proteins. A lower salt content gel contains weaker disulfide bonds, hydrophobic and electrostatic interactions and stronger hydrogen bonds. Partial replacement by KCl does not affect the chemical bonds. Calcium and magnesium chlorides enhance hydrophobic interactions and weaken hydrogen bonds and electrostatic interactions. Thus, a lower salt content weakens the gel and the presence of divalent salts solidifies the gel [125].

The reduction of the salt content in the range of 0.25–1.0% and the partial salt replacement by mixtures of 50% KCl, 25% MgCl_2_ and 25% CaCl_2_ in poultry sausages with a simultaneous salt and fat content reductions were tested by Schmidt et al. [126]. Additions of 0.5–1.0% collagen were used to adjust the physicochemical properties. The reformulation significantly changed the texture, water activity and pH of the sausages, but the sensory acceptability was maintained. The combination of 0.5% collagen, 0.25% salt substitute and 0.25% salt proved to be the best. The optimal addition ratio of 0.85% KCl and 0.25% polyphosphates was determined in the model emulsion. Then, sausages with 1.0–1.75% salt were made and their stability was monitored. Samples with a higher salt content were of better quality in most parameters; however, a 25% reduction in salt content was considered feasible [127]. Salt doses in the range of 0–2% were tested in fresh pork sausages. No statistically significant differences were found between the sausages with a salt content of 1; 1.5 and 2% in the total number of microorganisms, thiobarbiturate number, taste, texture and cooking loss. Consumers were able to distinguish between 1 and 2% salt content due to the taste [128]. In restructured chicken nuggets with NaCl replaced by CaCl_2_ the original salt content of 1.5% was reduced to 25–75%, maintaining the original ionic strength of the material. Sensory acceptance did not show big changes among original and tested products and what’s more, in samples with calcium chloride, the calcium content increased [129].

The 25% salt replacement with KCl, combined with the ripening of dry-cured lamb ham at high temperatures and the formation of volatile compounds, were studied by Luo et al. [130]. Higher temperature and salt reduction positively affected flavour formation, increased proteolysis and free amino acids content. Salt influenced proteolytic enzymes inhibition; therefore, in reduced-salt meat products, an increase in the proteolytic index was observed [47,131]. To reach the same safety condition of the reduced-salt dry-cured Italian ham, the cold-drying period was prolonged. Zhou et al. [132] salted the restructured bacon with 1.5% salt and 0–1.0% mixture of KCl and CaCl_2_ and then treated the samples with ultrasound at 600 W for 30 min. The mixture of 0.25% KCl and 0.25% CaCl_2_ significantly increased lipid and protein oxidation and free amino acids release. The sensory evaluation showed flavour improvement due to the formation of volatile compounds. Use of CaCl_2_ as a salt substitute was found to induce more lipid oxidation than the combination of NaCl and KCl [133].

Salting a ham from the bone using 1% alternative salting mixtures was tested by Lorenzo et al. [134]. The mixtures contained various proportions of potassium, calcium and magnesium salts: I—100% NaCl; II—50% NaCl and 50% KCl; III—45% NaCl, 25% KCl, 20% CaCl_2_ and 10% MgCl_2_; IV—30% NaCl, 50% KCl, 15% CaCl_2_ and 5% MgCl_2_. The highest content of microorganisms was found when using Mixture II. Hams containing Mixtures II and IV achieved the best sensory evaluation. Sensory changes of stewed ham with a salt content reduced from 3.4% to 1.4% and 25% potassium chloride replacement were also observed by Greiff et al. [135]. Furthermore, the salty taste, hardness and colour of the samples were found to change. Also, an aftertaste was found, but a 25% replacement of salt with potassium chloride caused only nonsignificant changes. Nielsen et al. [136] prepared and evaluated salmon pâtés with the addition of a natural mixture of mineral salt containing 30% of potassium (Saltwell^®^) and sodium chloride. The sodium content was reduced by 22%. The microbial activity was not affected and only small differences were found in texture, saltiness and fish flavour during the sensory analysis.

The authors Horita et al. [137] studied the effect of salt substitution on the quality of cheap sausages containing separated meat. A 25–50% of the salt was replaced by a mixture of chlorides containing calcium, potassium and sodium so that the ionic strength corresponded to 2% sodium chloride. When replacing the salt with 50% CaCl_2_, the meat released the most water and the resulting product was firm. What’s more, this product was not homogeneous in the cut; pores and cavities indicated poor stability of the emulsion. Sausages with a mixture of 25% CaCl_2_ and 25–50% KCl did not differ from the control ones in pH, water activity and colour. Sensory quality of sausages with a 50% salt content or with 25% CaCl_2_ and 25% KCl or with 50% KCl was rated worse than the quality of control sausages. Rios-Mera et al. [138] used micronized salt in the amount of 0.5–1.5% to salt beef burger. The burgers with 1% of micronized salt showed similar salty taste intensity and pH, colour, and yield as the samples of burgers with 1.5% of regular salt. The effect of partial salt replacement with KCl was recently studied on the example of dry-cured Xuanwei ham. Four samples were prepared with 30–60% salt dose replacement. As in other cases of salt replacement, the moisture of the samples increased compared to the control sample. Substances involved in the aroma of ham were analyzed by ultra-performance liquid chromatography-tandem mass spectrometry. Free amino acids and oleic acid were found to appear depending on the amount of KCl. In the sensory analysis, panelists rated as the best the ham sample with 30–40% KCl in the curing salt [139].

#### 3.3.2. Use of Flavour Enhancers

Salting of minced meat is carried out by simply mixing meat with the salting mixture, but salting of compact pieces of meat is a long-term process, because it is necessary to ensure perfect salt penetration into the meat. The salting of meat can be combined with the use of spices or seasoning extracts and also with flavour enhancers, for example, with sodium glutamate, inosine sodium, yeast extract, protein hydrolysates, lysine, alanine, taurine, glycine, guanidine salts or soy sauce [20,140]. Gaudette and Pietrasik [141] tested 2 salt substitutes and one flavour enhancer in ham and smoked turkey meat. Salt-replacement products were rated as less salty and more bitter, and the taste was enhanced in smoked meat. It is practically possible to use a combination of a salt substitute and an enhancer for sausages with a complex taste. This is more difficult in the meat itself, where the bitter taste has to be masked. Cold smoking process and smoking at 56 °C combined with replacement of salt by potassium chloride (25% and 50% KCl) were used for smoked salmon preparation. The salt content in the control sample was 5%. The salmon samples with 50% amount of KCl were more bitter than the control samples. The samples with 25% salt replacement were similar to the samples without salt reduction. The 2-week shelf-life of the new product was determined [142].

Four species of edible seaweed (red *Porphyra umbilicalis* and *Palmaria palmata*, brown *Himanthalia elongata* and *Undaria pinnatifida*) were used as a source of salty taste in reformulated Frankfurter sausages in the amount of 1%. In sausages, the salt content was reduced by 50%, the moisture and protein content increased and darker colour and softer texture were observed. The sensory profile and volatile compounds content were changed compared to control samples. Nevertheless, the algae *Himanthalia elongata* is considered promising for meat product reformulations [143].

Guinard et al. [144] used the taste properties of mushrooms as flavour enhancers and ingredients to mitigate the effects of salt reduction. The salt content of the mixed minced meat product was reduced by 25% and meat was flavoured with mushrooms. Differences were recorded between samples with full salt content, and no differences were found for samples with lower NaCl content according to the sensory assessors. The flour from edible mushroom *Agaricus bisporus* and *Pleurotus ostreatus* incorporated into Frankfurter sausages in the amount of 2.5 and 5% allowed a partial replacement of salt (50%) and also replacement of 30–50% of fat. The addition of mushroom flour increased moisture and fiber content and the colour, taste and texture were changed, but the level of 2.5 and 5% of mushroom flour was well applicable [145]. In Brazil, Horita et al. [146] tried to improve the sensory quality and microbial stability of cheap sausages with reduced salt content by adding variously treated garlic. Fresh garlic also acted as an antioxidant.

Low fat Bologna sausages were made with 50% salt substitution by KCl and additions of lysine or smoked liquid as flavour enhancers. Salt restriction did not affect physicochemical, technological or microbiological parameters. In the sensory profile the salty taste was limited and foreign tastes such as bitter, astringent and metal-like tastes were more pronounced. The addition of 1% lysine or 0.1% smoking fluid reduced aftertaste [147]. Vidal et al. [140] used the addition of amino acid lysine and yeast extract to minimize the negative sensory effects of salt substitutes KCl and CaCl_2_. Similarly, the authors da Silva et al. [148] prepared low-fat Bologna sausages with 60% replacement of NaCl by KCl and by addition of amino acids as flavour enhancers. The product with addition of 1% NaCl, 1.5% KCl, 1% arginine and 0.2% histidine was acceptable, because amino acids application compensated for the adverse effects of KCl.

The reduction of the salt content in the stewed ham to 1–1.2% using a modified thermal process and the addition of Palmaria palmata seaweed extract was tested by Barbieri et al. [149]. Algae extract did not affect the technological properties and the taste was positively affected. At the same time, however, the yield decreased by 5% and the products were drier after heat treatment.

Reformulation of the White pudding product in terms of fat and salt content was performed by Fellendorf et al. [150]. Samples with a fat content of 2.5–20% and a salt content of 0.2–1% were prepared. The product containing 0.8 and 1% salt was more acceptable as long as it did not contain too little fat. Products with significantly lower fat and salt content were rated as harder, little juicy, over-spiced and were of light to yellow colour. A product containing 0.6% salt and 15% fat was acceptable. In further modifications, the product with 10% fat and 0.6% sodium in the form of sodium citrate was well evaluated, as well as the combination of KCl with glycine. The samples were equally perceived as stiffer and more spicy.

An alternative could also represented by dairy products as salt substitutes, for example a low-mineral whey permeate from cheese production and a high-mineral milk permeate. They were added to products such as fish pâté or pudding. The low-mineral preparation improved the texture and water-binding activity and enabled to reduce the salt dose to 0.8%. In addition to the texture, the highly mineralized preparation also affected the taste of the product and can be used as a salt substitute [126]. The alternative seasoning based on pomace from red wine production was prepared by García et al. [151]. The new seasoning was used in beef patties stored in air and in patties packaged in a modified atmosphere. The application of 2% of seasoning inhibited the microbial contamination in low salted beef patties and increased the fiber and phenolic compounds content.

#### 3.3.3. Reduced Salt Content and Microorganisms Growth in Meat Product

By reducing the salt content in the meat products, the growth conditions of the microorganisms and also their diversity were changed. This fact is important especially for fermented meat products, where no heat treatment is used and the shelf-life of the products is ensured mainly by a pH decrease, reduction of water activity and appropriate salt content. In beef and pork salami, salt was replaced by KCl and CaCl_2_ and, the water activity and pH were changed. At a salt content lowered by 55% and salt substitution by KCl and CaCl_2_ mixture, the technological properties of the products did not change, but the taste, texture and overall acceptability of the products were changed [152]. Turkey sausages were used for testing of two commercial salt substitutes and one flavour enhancer blend. The water-binding activity of the meat and the texture were not disrupted, and the shelf-life at 60 days of cold storage was satisfactory. Sensory acceptability did not differ much, but one salt substitute caused aftertaste compared to the control sample [153]. Reducing the salt content from 2.2% to 1.1% and 0.55% in fermented sausages slowed down oxidative changes and aldehyde formation, but at the same time promoted proteolysis, carbonylation and Schiff bases formation. Sausages with reduced salt content were also firmer [154].

Microbiological and biochemical parameters were monitored in dried Portuguese sausages prepared with a lower salt dose. A significant effect on the number of microorganisms and the content of biogenic amines in the product was found. Slight changes also occurred in the composition of fatty acids. The texture of the less salty sausages was softer and sticky. The product with 3% salt had a more balanced taste compared to a control sample containing 6% salt [155,156].

Dry fermented sausages have their typical aroma given by the microflora adapted to the conditions used in meat production. As the salt content is reduced, the microflora composition of such products changes, and so does the aroma of the product. Some yeast strains were isolated and tested for aromatic substances formation. Selected strains were cultivated and added to the products [157]. In another study, dried fermented fat- and salt-reduced sausages were inoculated with *Debaromyces hansenii*. The salt content reduction was reflected in increased lipolysis and oxidation, and thus in rancid aroma. The reduction in fat content meant a shift of aroma to carbohydrate decomposition products. Yeast inoculation caused higher lipolysis and at the same time, a better antioxidant effect. In aroma, substances formed by the degradation of amino acids and fruit-flavored esters were pronounced. However, when the salt and fat contents decreased together, the effect of the yeast was not clear [158]. The sensory acceptability and functional properties of fermented sausages with reduced fat and salt levels were tested by Mora-Gallego et al. [159]. The addition of 0.64% KCl increased water activity, yield loss, caused the changes of textural properties such as hardness, gummity and cohesiveness, but, at the same time, the sensory impression changed minimally. The addition of sunflower oil managed to reduce the stiffness and cohesiveness of the product, but at the cost of higher friability and oily aftertaste.

Muchaamba et al. [160] partially replaced the salt by potassium lactate (2.8% NaCl and 1.6% potassium lactate) in salami and studied the growth of listeria and starter cultures during the salami ripening. The addition of low salt with potassium lactate showed similar anti-listeria effect as in the control sample. The growth of the starter culture was not significantly affected. The lethality of Salmonella in Biltong marinade depending on salt concentration and salt replacement was also tested. The alternative salts potassium chloride and calcium chloride were added into marinade in the concentration of 2.2%. Beef pieces were inoculated by Salmonella strains, tumbled in the marinade and then dried at 23.9 °C for 10 days. The number of Salmonella was reduced. It was shown that the use of salt substitutes in the marinade would allow the preparation of healthy and safe meat products with reduced salt content [161].

In many cases, reducing the salt content will cause an increase in water activity of the meat products and an increased risk of multiplication of pathogenic microorganisms. Experiments were performed to verify the microbial stability of less salty products or to directly test the survival of selected pathogens. For example, raw pork sausages with a salt content of 1.5 and 2% were prepared and stored for 21 days in a vacuum package or in modified atmosphere. Visible signs of spoilage were observed after 21 days. The microflora of sausages with 1.5% salt was more diverse than of sausages with 2% salt, so the salt content reduction resulted in faster spoilage and higher diversity of the microbial population [162]. In another experiment, a new spice preparation was developed based on wine pomace, which was added to beef with different salt content (1–2%). The meat was stored at 4 ° C in a modified atmosphere or in the presence of air. The addition of 2% of the preparation improved the microbiological stability of the meat, blocked the growth of mesophilic microorganisms and lactic acid bacteria. Furthermore, the content of potassium, calcium and phenolics increased in meat [151]. García-Díez et al. [163] investigated the possibility of *Listeria monocytogenes* survival in vacuum-packed chorizo salami with different salt, sugar and starter culture of *Lactobacillus sakei*. The addition of sugar and starter culture did not affect listeria. Higher salt content (1.5–3%) and storage at 22 °C reduced the number of listeria surviving in the product.

## 4. Special Technologies

In addition to the replacement of sodium chloride with other compounds, attempts are made to use new technologies that should facilitate the diffusion of salt into meat and thus allow even colouring of the product and distribution of salty taste. These are mainly experiments using high-pressure technology, which also inactivates vegetative forms of microorganisms. The effect of high pressure on the inactivation of *Listeria innocua* and *Enterococcus faecium* in marinated meat with reduced salt content and the effect on the physicochemical properties of meat were investigated by Rodrigues et al. [164,165]. The content of microorganisms was not affected by marinating alone, but by high pressure, which reduced the content of microorganisms by six orders of magnitude. Marination in a mixture of 2% salt and 2% citric acid was the most effective. Samples with higher acid content had lower pH and slower fat oxidation. The meat pressurized at 600 MPa was harder. The emulsified meat product served as a model for monitoring the effect of a combination of high pressure of 100–400 MPa, reduction of fat content to 20% and salt to 1%. The use of high pressure reduced cooking loss, changed the colour of the meat and reduced the solubility of actin and myosin [166]. The effect of high pressure (up to 300 MPa) and salt content in the range of 0–2.5% on the poultry meat quality before and after heat treatment was studied by Ros-Polski et al. [167]. The combined effect of salt and high pressure influenced pH but not water activity. The salt in low concentrations, together with the effect of high pressure, improved colour and texture of the meat. The effect of 0–1.9% salt, 0 and 0.25% phosphates and high pressure (100–600 MPa) in various production stages on the quality parameters of stewed ham was also monitored. Pascalization of the raw meat or meat after brining damaged the structure and water-binding activity of the meat; however, the pressure of 100 MPa applied after meat massaging had a positive effect. The salt content could be reduced to 1.1% using 0.2% KCl and a high-pressure treatment employed after meat massaging [168]. The effect of high pressure in combination with the use of sodium, potassium or calcium chloride in concentrations of 0–2.5% on the survival of Listeria monocytogenes in salted poultry meat was tested by Balamurugana et al. [169]. The high pressure of 100–300 MPa alone or in combination with salts was insufficient to inactivate listeria, but the pressure of 600 MPa was already effective. The use of salt or KCl was less effective on listeria than the use of calcium chloride.

Ultrasound technology is used primarily to accelerate the process of salt diffusion into meat products such as ham, bacon or chicken. Ultrasound can also be used to modify the texture of meat and increase the water-binding activity. It can be included in the brining and marinating, cooking or tumbling phases. The low-intensity ultrasound (LIU with frequencies 20–100 kHz and intensities < 1 W/cm^2^) and high-intensity ultrasound (HIU with frequencies >100 kHz and intensities > 1 W/cm^2^) can be used. The LIU is a non-invasive, inexpensive and easier method to use. The HIU changes the physicochemical properties and texture of the meat, creates acoustic cavitation and sponge effect due to the compression and decompression of meat. The disadvantage of ultrasound is the possibility of lipids’ accelerated oxidation and thus the reduction of the shelf life of meat products. The matrix effect is strongly applied during the ultrasound application, therefore the parameters of the equipment have to be tested and adjusted for each processed product [170].

Experiments were also performed using ultrasound [171] for better diffusion of salt or salt substitutes during pork brining. It was shown that the effect of ultrasound is small; the penetration and diffusion of salt into the meat was improved slightly only at the highest process intensity. The effect of ultrasound application on the properties of restructured cooked ham with reduced salt content was tested. The ham samples with 0.75–1.5% of salt were treated by ultrasound at nominal current of 600 W/cm^2^ for 10 min. The ultrasound application increased the hardness of ham samples, decreased fluid release and caused myofibrillar damage, but the sensory acceptance of ham with 0.75% of salt was improved [172].

Rizo et al. [173] developed a new smoke-flavoured and salt-reduced salmon product with the use of water vapour permeable bags. Salmon meat was salted and vacuum-packed into vapour permeable bags. Packaged meat was smoked in a drying chamber. The 37% sodium replacement by KCl or commercial sodium-free salt application in new smoke-flavoured salmon products did not cause any impairment of sensory properties or shelf life. Barnett et al. [60] tried to process chicken pasta meal with microwave-assisted thermal sterilization (MATS) and found out that over longer storage time at ambient temperature meals processed by MATS increased their aroma, taste, and flavour intensities.

Ultrasound and basic electrolyzed water were tested as a treatment of meat emulsions to reduce the salt content. An ultrasound bath at 25 kHz, 175 W in normal mode operating for 0–20 min was applied. After 20 min of sonication in the meat emulsion with 10–20% salt reduction the emulsion stability, cooking yield and texture were improved. The meat emulsion treated with basic electrolyzed water showed good stability and fat retention, and moderate water retention. The combination of both methods was recommended for the meat emulsions with up to 30% salt reduction [174]. Bhat et al. [175] tested the application of pulsed electric field for beef jerky treatment. The salt level of 2% was reduced to 1.2%. Pulsed electric field affected shear force and toughness of meat; however, colour, yield, oxidative and microbial stability were not changed. This treatment improved salt diffusion into meat and saltiness perception of jerky meat.

Kim et al. 2021 [176] included irradiation technology among the new technologies, which should extend the shelf life of meat. However, the results of the experiments are controversial, because of undesired sensory changes of the irradiated meat occurring due to the lipid and proteins oxidation.

The use of microbial transglutaminase was also tested to increase water-binding activity. The addition of transglutaminase improved the product structure and resulted in cross-links creation, thus increasing the cohesion and strength of the myofibrillar protein gel. Similarly, a restructured fish meat product with new functional properties, without bones, suitable for children or the elderly with hypertension, was made. Microbial transglutaminase was also used to modify the texture, with 1.5% salt content and an enzyme dose of 300 U/kg. The optimal process temperature of 25 °C and the enzyme dose of 377 U/kg were determined. This product was then usable, e.g., for sandwiches [177,178].

## 5. Conclusions

High daily salt intake poses a significant risk to human health. The reduction of salt content in basic foods is therefore very necessary, but it has a negative effect on the technology of food production. The salt in foods is usually replaced by potassium, magnesium and calcium salts, and these salts are supplemented with amino acids, protein hydrolysates, sodium glutamate, nucleotides, spices and herbs. These salt substitutes can be used only to a certain extent, because the products with a higher substitution level (usually over 30% of the salt dose) are less acceptable to consumers.

In cheeses, the main problems are higher water activity and the changes in the microbial population in favor of less salt-tolerant microorganisms. Salt content reduction promotes the growth of lactobacilli, but also of unwanted microflora. It is, therefore, necessary to optimize the composition of starter cultures. The use of probiotic cultures increases the resistance of cheeses to spoilage. As the residual lactose content decreases and the activity of proteolytic enzymes increases, lower peptides and free amino acids are released. These substances significantly affect the aroma of the cheeses. It is likely that by salt content reduction, new products will be created that differ from the original cheeses in hardness, strength and aroma.

In bakery products, salt affects the development of gluten and its viscoelastic properties. The presence of salt improves the flexibility and ability to the mechanical processing of dough. The dough with reduced salt content is less stable, prone to overmixing, weak and sticky. Reduction of the salt content promotes the rate of yeast fermentation and enzyme activity, but the content of residual sugars decreases. Low sugar level decreases the intensity of the Maillard reaction and the formation of a dark crust on the surface of the pastry, while the formation of acrylamide also decreases. The three-dimensional network of gluten is weaker, the volume of bread decreases, and the crumb has irregular pores. Baked bread is dry and crumbly. As the fermentation time is extended, the pore size increases and the salty taste is perceived more intensely. To enhance the salty taste in breads with salt replacements, it is possible to add salt enhancers or use the classic procedure with sourdough preparation. Fermentation produces a number of aromatic substances, including GABA and MSAG. However, the addition of dried sourdough prolongs the mixing and leavening time. Enhancement of the salty taste perception is also possible with encapsulated salt. 

Salt in meat products regulates the solubility of myofibrillar proteins and affects the ability of meat proteins to bind free water and fat. Salt is the carrier of nitrite and spice extracts in meat production. Products with reduced salt content are often flavoured with salt substitutes or salt enhancers, and the addition of polyphosphates ensures sufficient water binding. When modifying the texture, monovalent metals soften the gel of myofibrillar proteins, and divalent metals strengthen this gel. Additions of collagen, plant proteins and hydrocolloids, including fungi and seaweed, are also used to modify the texture. To improve the cohesiveness of meat products, the application of microbial transglutaminase is used, which is able to combine pieces of meat into a cohesive mass. As with cheeses, water activity is increasing and this increase will allow a wider range of microorganisms to grow. This is especially important in the production of fermented meat products, where the product is acidified more quickly. Modern technologies such as ultrasound, high-pressure treatment and pulsed electric field have been tested for better penetration of salt into the meat.

In all modifications of recipes of food products with a reduced salt dose, it is necessary to ensure not only health and safety but also consumer acceptability in terms of texture, appearance and taste. This is the biggest challenge facing food reformulations.

## Figures and Tables

**Table 1:** The nutrition claims related to sodium and salt content [17].

Nutrition Claims	g Na/100 g	g NaCl/100 g
Low sodium/salt	0.12	0.305
Very low sodium/salt	0.04	0.102
Sodium-free or salt-free	0.005	0.013

**Table 2:** The overview of materials applicable as salt substitutes and salt enhancers, created by authors.

Salt Taste Sources	Salt Taste Enhancers
modified sodium chloride	plants as a salty taste enhancer
micronized salt	herbs and spices
encapsulated salt with lipids	extracts of herbs or spices
modified shape of salt crystals	seaweed
crystal aggregates	
hollow salt crystals	
salt nanoparticles on polysaccharide carriers	
organic and inorganic salts	amino acids and peptides
potassium chloride	glycine
magnesium chloride	lysine
ammonium chloride	arginine
calcium chloride, calcium carbonate	ornithine
potassium citrate and diphosphate	histidine
magnesium and sodium sulphate	plant protein hydrolysate
	yeast extracts
plants as a salt substitutes	other enhancers
seaweed	trehalose
halophytes	lactic acid salts
	glutamates
	adenosine-5’-monophosphate
	milk permeate
	sourdough
	edible mushrooms

## Data Availability

Not applicable.

## References

[1] Guido D., Perna S., Carrai M., Barale R., Grassi M., Rondanelli M. (2016). Multidimensional evaluation of endogenous and health factors affecting food preferences, taste and smell perception. J. Nutr. Health Aging.

[2] Hardikar S., Hochenberger R., Villringer A., Ohla K. (2017). Higher sensitivity to sweet and salty taste in obese compared to lean individuals. Appetite.

[3] Turck D., Castenmiller J., de Henauw S., Hirsch-Ernst K.-I., Kearney J., Knutsen H.K., Maciuk A, Mangelsdorf I., McArdle H.J., EFSA NDA Panel (EFSA Panel on Nutrition, Novel Foods and Food Allergens) (2019). Scientific Opinion on the dietary reference values for sodium. EFSA J..

[4] Grillo A., Salvi L., Coruzzi P., Salvi P., Parati G. (2019). Sodium intake and hypertension. Nutrients.

[5] Burnier M., Phan O., Wang Q. (2007). High salt intake: A cause of blood pressure-idependent left ventricular hypertrophy?. Nephrol. Dial. Transplant..

[6] Rodriguez C.J., Bibbins-Domingo K., Jin Z., Daviglus M.L., Goff D.C., Jacobs D.R. (2011). Association of sodium and potassium intake with left ventricular mass: Coronary artery risk development in young adults. Hypertension.

[7] World Health Organization (2007). Reducing Salt Intake in Population.

[8] Thout S.R., Santos J.A., McKenzie B., Trieu K., Johnson C., McLean R., Arcand A.A., Campbell N.R.C., Webster J. (2019). The Science of Salt: Updating the evidence on global estimates of salt intake. J. Clin. Hypertens..

[9] Mallamaci F., Tripepi G. (2017). Salt restriction in chronic kidney disease: A simple need or a must?. Kidney Blood Press Res..

[10] Ohta Y., Tsuchihashi T., Kiyohara K., Oniki H. (2013). High salt intake promotes a decline in renal function in hypertensive patients: A 10-year observational study. Hypertens. Res..

[11] D’Elia L., Rossi G., Ippolito R., Cappuccio F.P., Strazzullo P. (2012). Habitual salt intake and risk of gastric cancer: A meta-analysis of prospective studies. Clin. Nutr..

[12] Shikata K., Kiyohara Y., Kubo M., Yonemoto K., Ninomiya T., Shirota T., Tanizaki Y., Doi Y., Tanaka K., Oishi Y. (2006). A prospective study of dietary salt intake and gastric cancer incidence in a defined Japanese population: The Hisayama study. Int. J. Cancer.

[13] He F.J., Tan M., Ma Y., MacGregor G.A. (2020). Salt Reduction to Prevent Hypertension and Cardiovascular Disease: State of the Art Review. J. Am. Coll. Cardiol..

[14] Trusková I., Bezegová D., Horváthová-Trúchla K., Jóžeffiová E., Kromerová K., Némethová D. (2016). Best Practices of the Member States in Food Reformulation.

[15] World Health Organization (2021). WHO Global Sodium Benchmarks for Different Food Categories.

[16] Web of Science. https://www.webofscience.com/wos/woscc/advanced-search.

[17] Regulation (EC) No 1924/2006 of the European Parliament and of the Council of 20 December 2006 on Nutrition and Health Claims Made on Foods. https://eur-lex.europa.eu/legal-content/EN/TXT/PDF/?uri=CELEX:32006R1924&from=CS.

[18] Bansal V., Mishra S.K. (2020). Reduced-sodium cheeses: Implications of reducing sodium chloride on cheese quality and safety. Compr. Rev. Food Sci. Food Saf..

[19] Nejedlá M. Sociálně marketingové nástroje ke snížení spotřeby soli [Social marketing tools to reduce salt consumption]. Proceedings of the BCA—Bilateral Collaborative Agreements. Konference Školní Stravování [School Catering Conference].

[20] Chen J., Hu Y., Wen R., Liu Q., Chen Q., Kong B. (2019). Effect of NaCl substitutes on the physical, microbial and sensory characteristics of Harbin dry sausage. Meat Sci..

[21] Opletal L., Wimmer Z., Čopíková J., Lapčík O., Moravcová J., Cahlíková L., Drašar P. (2011). Slaná chuť přírodních látek a jejich derivátů [Salty taste of natural substances and their derivatives]. Chem. Listy.

[22] Rama R., Chiu N., da Silva M.C., Hewson L., Hort J., Fisk I.D. (2013). Impact of salt crystal size on in-mouth delivery of sodium and saltiness perception from snack foods. J. Texture Stud..

[23] Micronized Salt Market to Record 3% Growth on Back of Reduced Salt Consumption. https://ksusentinel.com/2021/05/12/food-grade-micronized-salt-market-to-record-3-growth-on-back-of-reduced-salt-consumption-fact-mr/.

[24] Lake Shore Gazette Micronized Salt Market Will Reach US $3305.8 Mn by 2018 to 2028: Persistence Market Research. https://www.lakeshoregazette.com/2021/08/16/micronized-salt-market-will-reach-us-3305-8-mn-by-2018-to-2028persistence-market-research/.

[25] Vinitha K., Leena M.M., Moses J.A., Anandharamakrishnan C. (2021). Size-dependent enhancement in salt perception: Spraying approaches to reduce sodium content in foods. Powder Technol..

[26] Sun C., Zhou X., Hu Z., Lu W., Zhao Y., Fang Y. (2020). Food and salt structure design for salt reducing. Innov. Food Sci. Emerg. Technol..

[27] Rios-Mera J.D., Selani M.M., Patinho I., Saldana E., Contreras-Castillo C.J. (2021). Modification of NaCl structure as a sodium reduction strategy in meat products: An overview. Meat Sci..

[28] Hurst K.E., Ayed Ch., Derbenev I.N., Hewson L., Fisk I.D. (2021). Physicochemical design rules for the formulation of novel salt particles with optimised saltiness. Food Chem..

[29] (2014). Review of Current Salt Replacing Ingredients. Campden BRI. https://www.campdenbri.co.uk/white-papers/salt-replacing.php.

[30] Reducing Global Sodium Intake: Innovative Ingredient Solutions (TATE&LYLE Corporate Literature). https://www.tateandlyle.com/ingredient/soda-lo-salt-microspheres.

[31] Ayyash M., Sherkat F., Shan N., Preedy V.R., Watson R.R., Patel V.B. (2013). Sodium Chloride Substitution of Cheese. Handbook of Cheese in Health.

[32] Bae I., Park J.H., Choi H.Y., Jung H.K. (2017). Emerging Innovations to Reduce the Salt Content in Cheese; Effects of Salt on Flavor, Texture, and Shelf Life of Cheese; and Current Salt Usage: A Review. Korean J. Food Sci Anim. Resour..

[33] Loudiyi M., Aït-Kaddour A. (2019). Evaluation of the effect of salts on chemical, structural, textural, sensory and heating properties of cheese: Contribution of conventional methods and spectral ones. Crit. Rev. Food Sci. Nutr..

[34] Roginski H., Foquay J.W., Fox P.F. (2002). Encyclopedia of Dairy Sciences.

[35] Matz S.A. (2012). Snack Food Technology.

[36] Šedivý P., Dostál J., Kovaříková D., Martinek V. (2013). Bakery Technology I.

[37] Jekle M., Beck M., Becker T. (2014). Sodium content in baked products. Baking+Biscuit.

[38] Diler G., Le-Bail A., Chevallier S. (2016). Salt reduction in sheeted dough: A successful technological approach. Food Res. Int..

[39] Monteiro A.R.G., Nakagawa A., Pimentel T.C., Sousa I. (2021). Increasing saltiness perception and keeping quality properties of low salt bread using inhomogeneous salt distribution achieved with salt agglomerated by waxy starch. LWT.

[40] McCann T.H., Day L. (2013). Effect of sodium chloride on gluten network formation, dough microstructure and rheology in relation to breadmaking. J. Cereal Sci..

[41] Sluimer P. (2005). Principles of Breadmaking. Functionality of Raw Materials and Process Steps.

[42] Silow C., Zannini E., Axel C., Lynch K.M., Arendt E.K. (2016). Effect of salt reduction on wheat-dough properties and quality characteristics of puff pastry with full and reduced fat content. Food Res. Int..

[43] Belz M.C.E., Ryan L.A.M., Arendt E.K. (2012). The Impact of Salt Reduction in Bread: A Review. Crit. Rev. Food Sci. Nutr..

[44] Salovaara H. (2009). Salt in Bread: Technical, Taste and other Parameters for Healthy Eating.

[45] Cobos Á., Díaz O., Cheung P. (2014). Chemical Composition of Meat and Meat Products. Handbook of Food Chemistry.

[46] Jeong J.Y. (2017). Effects of Short-Term Presalting and Salt Level on the Development of Pink Color in Cooked Chicken Breasts. Korean J. Food Sci. Animal..

[47] Schivazappa C., Virgili R. (2020). Impact of salt levels on the sensory profile and consumer acceptance of Italian dry-cured ham. J. Sci. Food Agric..

[48] Sebranek J.G. An Overview of Functional Non-Meat Ingredients in Meat Processing: The Current Toolbox. Proceedings of the American Meat Science Association, 68th Annual Reciprocal Meat Conference, University of Nebraska-Lincoln.

[49] Desmond E. (2006). Reducing salt: A challange for the meat industry. Meat Sci..

[50] Kameník J., Zdolec N. (2017). Hurdle Technologies in Fermented Meat Production. Fermented Meat Products Health Aspects.

[51] Regulation (EC) No 1333/2008 Of the European Parliament and of the Council of 16 December 2008 on Food Additives, Annex II. https://eur-lex.europa.eu/legal-content/EN/TXT/PDF/?uri=CELEX:32008R1333&from=en.

[52] Ghawi S.K., Rowland I., Methven L. (2014). Enhancing consumer liking of low salt tomato soup over repeated exposure by herb and spice seasonings. Appetite.

[53] Kohri T., Kira R., Myojin C., Kawanishi M., Tomotake H. (2020). Enhancing effects of herbs on the salty taste perception of saline. J. Nutr. Vitaminol..

[54] Rysová J., Houška M., Novotná P., Laknerová I., Strohalm J. (2020). The application of *Ocimum sanctum* into the foods. Úroda.

[55] Rysová J., Laknerová I., Houška M., Novotná P., Strohalm J. (2017). The antioxidant activity of herbal homogenate extracts. Úroda.

[56] Krofta K., Houška M., Mikyška A., Čermák P. (2018). Changes in the composition of hop secondary metabolites induced by high hydrostatic pressure. J. Inst. Brew..

[57] Taladrid D., Laguna L., Bartolome B., Moreno-Arribas V. (2020). Plant-derived seasonings as sodium salt replacers in food. Trends Food Sci. Technol..

[58] Petropoulos S.A., Karkanis A., Martins N., Ferreira I.C.F.R. (2018). Edible halophytes of the Mediterranean basin: Potential candidates for novel food products. Trends Food Sci. Technol..

[59] Barreira L., Resek E., Rodrigues M.J., Rocha M.I., Pereira H., Bandarra N., da Silva M.M., Varela J., Custodio L. (2017). Halophytes: Gourmet food with nutritional health benefits?. J. Food Comp. Anal..

[60] Barnett S.M., Sablani S.S., Tang J., Ross C.F. (2019). Utilizing Herbs and Microwave-Assisted Thermal Sterilization to Enhance Saltiness Perception in a Chicken Pasta Meal. J. Food Sci..

[61] Patel S. (2016). *Salicornia*: Evaluating the halophytic extremophile as a food and a pharmaceutical candidate. Biotech.

[62] Mishra A., Patel M.K., Jha B. (2015). Non-targeted metabolomics and scavenginactivity of reactive oxygen species reveal the potential of *Salicornia brachiata* as a functional food. J. Funct. Foods.

[63] Silybaeva B.M., Mussabayeva B.K., Zharykbaksova K.S., Kydyrmoldina A.S., Kaygusuz O. (2016). Biologically Active Agents of *Salicornia europaea* L. Grown in East Kazakhstan. Res. J. Pharm. Biol. Chem. Sci..

[64] Elsebaie E.M., Elsanat S.Y.A., Gouda M.S., Elnemr K.M. (2013). Studies on antimicrobial and antioxidant efficiency of glasswort (*Salicornia fruticosa*) herb juice and methanolic extract in minced beef. Int. J. Mod. Agric..

[65] Lim S.J., Kim H.W., Hwang K.E., Song D.H., Kim Y.J., Ham Y.K., Jang S.J., Lee C.H., He F.Y., Choi Y.S. (2015). Effects of Glasswort (*Salicornia herbacea* L.) Hydrates on Quality Characteristics of Reduced-salt, Reduced-fat Frankfurters. Korean J. Food Sci. An..

[66] Lopes M., Cavaleiro C., Ramos F. (2017). Sodium Reduction in Bread: A Role for Glasswort (*Salicornia ramosissima* J. Woods). Compr. Rev. Food Sci. Food Saf..

[67] Joo S.Y., Choi H.Y. (2014). Antioxidant Activity and Quality Characteristics of Pork Patties Added with Saltwort (*Salicornia herbacea* L.) Powder. J. Korean Soc. Food Sci. Nutr..

[68] Nayak B., Roy S., Roy M., Mitra A., Karak K. (2018). Phytochemical, Antioxidant and Antimicrobial Screening of *Suaeda maritima* L (Dumort) against Human Pathogens and Multiple Drug Resistant Bacteria. Indian J. Pharm. Sci..

[69] Serrano C., Sapata M., Oliveira M.C., Gerardo A., Viegas C. (2020). Encapsulation of oleoresins for salt reduction in food. Acta Sci. Pol. Technol. Aliment..

[70] Onipchenko N., Doležalová M., Procházková E., Martinková I., Hrabě J. (2012). Změny mikroflóry během výroby pařených sýrů (Changes in the microflora during the production of steamed cheeses). Mlékařské Listy.

[71] Taylor T.M., Lathrop A.A. (2015). Evaluation of antimicrobials and salt replacers for use in low-sodium dairy products. J. Food Saf..

[72] McCarthy C.M., Wilkinson M.G., Kelly P.M., Guinee T.P. (2016). Effect of salt and fat reduction on proteolysis, rheology and cooking properties of Cheddar cheese. Int. Dairy J..

[73] McCarthy C.M., Wilkinson M.G., Kelly P.M., Guinee T.P. (2015). Effect of salt and fat reduction on the composition, lactose metabolism, water activity and microbiology of Cheddar cheese. Dairy Sci. Technol..

[74] Lu Y., Mc Mahon D.J. (2015). Effects of sodium chloride salting and substitution with potassium chloride on whey expulsion of Cheddar cheese. J. Dairy Sci..

[75] Murtaza M.A., Huma N., Sameen A., Murtaza M.S., Mahmood S., Mueen-ud-Din G., Meraj A. (2014). Texture, flavor, and sensory quality of buffalo milk Cheddar cheese as influenced by reducing sodium salt content. J. Dairy Sci..

[76] McMahon D.J., Oberg C.J., Drake M.A., Farkye N., Moyes L.V., Arnold M.R., Ganesan B., Steele J., Broadbent J.R. (2014). Effect of sodium, potassium, magnesium, and calcium salt cations on pH, proteolysis, organic acids, and microbial populations during storage of full-fat Cheddar cheese. J. Dairy Sci..

[77] Khetra Y., Kanawjia S.K., Puri R. (2016). Selection and optimization of salt replacer, flavour enhancer and bitter blocker for manufacturing low sodium Cheddar cheese using response surface methodology. LWT Food Sci. Technol..

[78] Ganesan B., Brown K., Irish D.A., Brothersen C., McMahon D.J. (2014). Manufacture and sensory analysis of reduced- and low-sodium Cheddar and Mozzarella cheeses. J. Dairy Sci..

[79] Jesus A.L.T., Fernandes M.S., Kamimura B.A., Silva L., Silva R., Esmerino E.A., Cruz A.G., Sant´Ana A.S. (2016). Growth potential of *Listeria monocytogenes* in probiotic cottage cheese formulations with reduced sodium content. Food Res. Int..

[80] Justia Patents (2010). Reduced-Salt Dairy Product with Improved Taste. Inventors: Frank Richard Meijer, Catharina Houdijk, Cornelis Muijlwijk, Neeltje Corine Oorschot Van: NIXON & VANDERHYE, PC Assignees: Origin: ARLINGTON, VA US IPC8. Class: AA23C1512FI. USPC Class: 426 37. U.S. Patent.

[81] Soltani M., Guzeler N., Hayloglu A.A. (2015). The influence of salt concentration on the chemical, ripening and sensory characteristics of Iranian white cheese manufactured by UF-Treated milk. J. Dairy Res..

[82] Kamleh R., Olabi A., Toufeili I., Daroub H., Younis T., Ajib R. (2015). The effect of partial substitution of NaCl with KCl on the physicochemical, microbiological and sensory properties of Akkawi cheese. J. Sci. Food Agric..

[83] Gandhi A., Shah N.P. (2016). Salt Reduction in a Model High-Salt Akawi Cheese: Effects on Bacterial Activity, pH, Moisture, Potential Bioactive Peptides, Amino Acids, and Growth of Human Colon Cells. J. Food Sci..

[84] Arboatti A.S., Olivares M.L., Sabbag N.G., Costa S.C., Zorrilla S.E., Sihufe G.A. (2014). The influence of sodium chloride reduction on physicochemical, biochemical, rheological and sensory characteristics of Mozzarella cheese. Dairy Sci. Technol..

[85] Henneberry S., O’Sulivan M.G., Kilcawley K.N., Kelly P.M., Wilkinson M.G., Guinee T.P. (2016). Sensory quality of unheated and heated Mozzarella-style cheeses with different fat, salt and calcium levels. Int. J. Dairy Technol..

[86] Henneberry S., Wikinson M.G., Kilcawley K.N., Kelly P.M., Guinee T.P. (2015). Interactive effects of salt and fat reduction on composition, rheology and functional properties of mozzarella-style cheese. Dairy Sci. Technol..

[87] Soares C., Fernando A.L., Alvarenga N., Martins A.P.L. (2016). Substitution of sodium chloride by potassium chloride in So Joo cheese of Pico Island. Dairy Sci. Technol..

[88] Dugat-Bony E., Sarthou A.S., Perello M.C., de Revel G., Bonnarme P., Helinck S. (2016). The effect of reduced sodium chloride content on the microbiological and biochemical properties of a soft surface-ripened cheese. J. Dairy Sci..

[89] Dugat-Bony E., Bonnarme P., Fraud S., Catelotte J., Sarthou A.S., Loux V., Rué O., Bel N., Chuzeville S., Helinck S. (2019). Effect of sodium chloride reduction or partial substitution with potassium chloride on the microbiological, biochemical and sensory characteristics of semi-hard and soft cheeses. Food Res. Int..

[90] Luzzi G., Brinks E., Fritsche J., Franz C.M.A.P. (2021). Effect of reduction of sodium content on the microbial ecology of Edam cheese samples. AMB Express.

[91] Tidona F., Bernardi M., Francolino S., Ghiglietti R., Hogenboom J.A., Locci F., Zambrini V, Carminati D, Giraffa G. (2019). The impact of sodium chloride reduction on Grana-type cheese production and quality. J. Dairy Res..

[92] Moatsou G., Zoidou E., Choundala E., Koutsaris K., Kopsia O., Thergiaki K., Sakkas L. (2019). Development of Reduced-Fat, Reduced-Sodium Semi-Hard Sheep Milk Cheese. Foods.

[93] Silva H.L.A., Balthazar C.F., Silva R., Vieira A.H., Costa R.G.B., Esmerino E.A., Freitas M.Q., Cruz A.G. (2018). Sodium reduction and flavor enhancer addition in probiotic prato cheese: Contributions of quantitative descriptive analysis and temporal dominance of sensations for sensory profiling. J. Dairy Sci..

[94] Ehsannia S., Sanjabi M.R. (2016). Physicochemical, microbiological and spoilage analysis of probiotic processed cheese analogues with reduced emulsifying salts during refrigerated storage. J. Food Sci. Technol..

[95] Pasqualone A., Caponio F., Pagani M.A., Summo C., Paradiso V.M. (2019). Effect of salt reduction on quality and acceptability of durum wheat bread. Food Chem..

[96] Sun X., Scanlon M.G., Guillermic R.M., Belev G.S., Webb M.A., Aritan S., Nickerson M.T., Koksel F. (2020). The effects of sodium reduction on the gas phase of bread doughs using synchrotron X-ray microtomography. Food Res. Int..

[97] Pflaum T., Konitzer K., Hofmann T., Koehler P. (2013). Influence of Texture on the Perception of Saltiness in Wheat Bread. J. Agric. Food Chem..

[98] Lobo C.P., Ferreira T.A.P.C. (2021). Hedonic thresholds and ideal sodium content reduction of bread loaves. Food Res. Int..

[99] El Ati J., Doggui R., El Ati-Hellal M. (2021). A Successful Pilot Experiment of Salt Reduction in Tunisian Bread: 35% Gradual Decrease of Salt Content without Detection by Consumers. Int. J. Environ. Res. Public Health.

[100] Israr T.R.A., Sohail M., Rashid S., Shehzad A. (2016). Salt reduction in baked products: Strategies and constraints. Trends Food Sci. Technol..

[101] Miller R., Jeong J. (2014). Sodium Reduction in Bread Using Low-Sodium Sea Salt. Cereal Chem..

[102] Kovac B., Knific M. (2016). The perception of low-salt bread among preschool children and the role of educational personnel in creating a positive attitude towards reformulated food. Zdr. Varst..

[103] Sinesio F., Raffo A., Peparaio M., Moneta E., Saggia Civitelli E., Narducci V., Turfani V., Ferrari Nicoli S., Carcea M. (2019). Impact of sodium reduction strategies on volatile compounds, sensory properties and consumer perception in commercial wheat bread. Food Chem..

[104] Quilez J., Salas-Salvado J. (2012). Salt in bread in Europe: Potential benefits of reduction. Nutr. Rev..

[105] La Croix K.W., Fiala S.C., Colonna A.E., Durham C.A., Morrissey M.T., Drum D.K., Kohn M.A. (2015). Consumer detection and acceptability of reduced-sodium bread. Public Health Nutr..

[106] Sayar S., Erdoglu F., Eydemir G., Nayman E. (2016). Partial substitution of sodium chloride by potassium chloride in bread: Effect on dough and bread properties. Qual. Assur. Saf. Crop. Foods.

[107] Spina A., Brighina S., Muccilli S., Mazzaglia A., Rapisarda P., Fallico B., Arena E. (2015). Partial Replacement of NaCl in Bread from Durum Wheat (*Triticum turgidum* L. subsp. *durum* Desf.) with KCl and Yeast Extract: Evaluation of Quality Parameters During Long Storage. Food Bioprocess Technol..

[108] Bassett M.N., Perez-Palacios T., Cipriano I., Cardoso P., Ferreira I., Samman N., Pinho O. (2014). Development of bread with nacl reduction and calcium fortification: Study of its quality characteristics. J. Food Qual..

[109] Kaur A., Bala R., Singh B., Rehal J. (2011). Effect of Replacement of Sodium Chloride with Mineral salts on Rhoelogical characteristics of Wheat Flour. Am. J. Food Technol..

[110] Arena E., Muccilli S, Mazzaglia A., Giannone V., Brighina S., Rapisarda P., Fallico B., Allegra M., Spina A. (2020). Development of Durum Wheat Breads Low in Sodium Using a Natural Low-Sodium Sea Salt. Foods.

[111] Li Y.L., Han K.N., Feng G.X., Wan Z.L., Wang G.S., Yang X.Q. (2021). Salt reduction in bread via enrichment of dietary fiber containing sodium and calcium. Food Funct..

[112] Nogueira A.D., Kussano J.T., Steel C.J. (2015). Sourdough reduces sodium in wheat flour doughs. Int. J. Food Sci. Technol..

[113] Voinea A., Stroe S.G., Codină G.G. (2020). The Effect of Sodium Reduction by Sea Salt and Dry Sourdough Addition on the Wheat Flour Dough Rheological Properties. Foods.

[114] Gobbetti M., De Angelis M., Di Cagno R., Calasso M., Archetti G., Rizzello C.G. (2019). Novel insights on the functional/nutritional features of the sourdough fermentation. Int. J. Food Microbiol..

[115] Valerio F., Conte A., Di Biase M., Lattanzio V.M.T., Lonigro S.L., Padalini L., Pontonio E., Lavermiococca P. (2017). Formulation of yeast-leavened bread with reduced salt content by using a Lactobacillus plantarum fermentation product. Food Chem..

[116] Penas E., Diana M., Frias J., Quilez J., Martinez-Villaluenga C.E. (2015). A multistrategic approach in the development of sourdough bread targeted towards blood pressure reduction. Plant Foods Human Nutr..

[117] Noguieira A.D., Steel C.J. (2016). Process Parameters and Technological Quality of French Rolls Produced with Dry Sourdough to Reduce Sodium. Cereal Chem..

[118] Zhao C.J., Kinner M., Wismer W., Ganzle M.G. (2015). Effect of Glutamate Accumulation During Sourdough Fermentation with Lactobacillus reuteri on the Taste of Bread and Sodium-Reduced Bread. Cereal Chem..

[119] Jimenez-Maroto L.A., Sato T., Rankin S.A. (2013). Saltiness potentiation in white bread by substituting sodium chloride with a fermented soy ingredient. J. Cereal Sci..

[120] Ambrisewicz-Walacik M., Tanska M., Rotkiewicz D., Pietak A. (2016). Effect of Various Sodium Chloride Mass Fractions on Wheat and Rye Bread Using Different Dough Preparation Techniques. Food Technol..

[121] Rodrigues I., Gonçalves L.A., Carvalho F.A., Pires M., Rocha Y.J., Barros J.C., Carvalho L.T., Trindade M.A. (2020). Understanding salt reduction in fat-reduced hot dog sausages: Network structure, emulsion stability and consumer acceptance. Food Sci. Technol. Int..

[122] Delgado-Pando G., Fischer E., Allen P., Kerry J.P., O’Sullivan M.G., Hamill R.M. (2018). Salt content and minimum acceptable levels in whole-muscle cured meat products. Meat Sci..

[123] Carkcioglu E., Rosenthal A.J., Candogan K. (2016). Rheological and Textural Properties of Sodium Reduced Salt Soluble Myofibrillar Protein Gels Containing Sodium Tri-Polyphosphate. J. Texture Stud..

[124] Chen X., Li Y., Zhou R.Y., Liu Z.M., Lu F.Z., Lin H., Xu X.L., Zhou G.H. (2016). L-histidine improves water retention of heat-induced gel of chicken breast myofibrillar proteins in low ionic strength solution. Int. J. Food Sci. Technol..

[125] Zhang Z.Y., Yang Y.L., Tang Y.Z., Chen Y.J., You Y.Y. (2016). Chemical forces study of heat-induced myofibrillar protein gel as affected by partial substitution of NaCl with KCl, MgCl2 and CaCl2. CYTA J. Food.

[126] Schmidt M.M., Dornelles R.C.P., Vidal A.R., Fontoura A., Kubota E.H., Mello R.O., Kempka A.P., Demiate I.M. (2017). Development of cooked and smoked chicken sausage with reduced sodium and fat. J.Appl. Poult. Res..

[127] Yotsuyanagi S.E., Contreras-Castillo C.J., Haguiwara M.M.H., Cipolli K.M.V.A.B., Lemos A.L.S.C., Morgano M.A., Yamada E.A. (2016). Technological, sensory and microbiological impacts of sodium reduction in frankfurters. Meat Sci..

[128] Cluff M., Steyn H., Charmba G., Bothma C., Hugo C.J., Hugo A. (2016). The chemical, microbial, sensory and technological effects of intermediate salt levels as a sodium reduction strategy in fresh pork sausages. J. Sci. Food Agric..

[129] Barros J.C., Gois T.S., Pires M.A., Rodrigues I., Trindade M.A. (2019). Sodium reduction in enrobed restructured chicken nuggets through replacement of NaCl with CaCl_2_. J. Food Sci. Technol..

[130] Luo J., Nasiru M.M., Zhuang H., Zhou G., Zhang J. (2021). Effects of partial NaCl substitution with high-temperature ripening on proteolysis and volatile compounds during process of Chinese dry-cured lamb ham. Food Res. Int..

[131] Pinna A., Saccani G., Schivazappa C., Simoncini N., Virgili R. (2020). Revision of the cold processing phases to obtain a targeted salt reduction in typical Italian dry-cured ham. Meat Sci..

[132] Zhou Y., Wang Y., Pan Q., Wang X.X., Li P.J., Cai K.Z., Chen C.G. (2020). Effect of salt mixture on flavor of reduced-sodium restructured bacon with ultrasound treatment. Food Sci. Nutr..

[133] Nachtigall F.M., Vidal V.A.S., Pyarasani R.D., Domínguez R., Lorenzo J.M., Pollonio M.A.R., Santos L.S. (2019). Substitution effects of NaCl by KCl and CaCl_2_ on Lipolysis of Salted Meat. Foods.

[134] Lorenzo J.M., Bermudez R., Dominguez R., Guiotto A., Franco D., Purrinos L. (2015). Physicochemical and microbial changes during the manufacturing process of dry-cured lacon salted with potassium, calcium and magnesium chloride as a partial replacement for sodium chloride. Food Control.

[135] Greif K., Staurem C.J., Nordvi B., Rustad T. (2015). Novel utilization of milk-based ingredients in salt reduced fish pudding. LWT Food Sci. Technol..

[136] Nielsen T., Mihnea M., Båth K., Cunha S.C., Fereira R., Fernandes J.O., Gonçalves A., Nunes M.L., Oliveira H. (2020). New formulation for producing salmon pâté with reduced sodium content. Food Chem. Toxicol..

[137] Horita C.N., Messias V.C., Morgano M.A., Hayakawa F.M., Pollonio M.A.R. (2014). Textural, microstructural and sensory properties of reduced sodium frankfurter sausages containing mechanically deboned poultry meat and blends of chloride salts. Food Res. Int..

[138] Rios-Mera J.D., Saldaña E., Cruzado-Bravo M.L.M., Patinho I., Selani M.M., Valentin D., Contreras-Castillo C.J. (2019). Reducing the sodium content without modifying the quality of beef burgers by adding micronized salt. Food Res. Int..

[139] Ding Y., Wang G., Zou Y., Zhao Y., Ge C., Liao G. (2021). Evaluation of small molecular metabolites and sensory properties of Xuanwei ham salted with partial replacement of NaCl by KCl. Meat Sci..

[140] Vidal V.A.S., Santana J.B., Paglarini C.S., da Silva M.A.A.P., Freitas M.Q., Esmerino E.A., Cruz A.G., Pollonio M.A.R. (2020). Adding lysine and yeast extract improves sensory properties of low sodium salted meat. Meat Sci..

[141] Gaudette N.J., Pietrasik Z. (2017). The sensory impact of salt replacers and flavor enhancer in reduced sodium processed meats is matrix dependent. J. Sens. Stud..

[142] Muñoz I., Guàrdia M.D., Arnau J., Dalgaard P., Bover S., Fernandes J.O., Monteiro C., Cunha S.C., Gonçalves A., Nunes M.L. (2020). Effect of the sodium reduction and smoking system on quality and safety of smoked salmon (Salmo salar). Food Chem. Toxicol..

[143] Vilar E.G., Ouyang H., O’Sullivan M.G., Kerry J.P., Hamill R.M., O’Grady M.N., Mohammed H.O., Kilcawley K.N. (2020). Effect of salt reduction and inclusion of 1% edible seaweeds on the chemical, sensory and volatile component profile of reformulated frankfurters. Meat Sci..

[144] Guinard J.X., Miller A.M., Mills K., Wong T., Lee S.M., Sirimuangmoon C., Schaefer S.E., Drescher G. (2016). Consumer acceptance of dishes in which beef has been partially substituted with mushrooms and sodium has been reduced. Appetite.

[145] Cerón-Guevara M.I., Rangel-Vargas E., Lorenzo J.M., Bermúdez R., Pateiro M., Rodríguez J.A., Sánchez-Ortega I., Santos E.M (2020). Reduction of Salt and Fat in Frankfurter Sausages by Addition of *Agaricus bisporus* and *Pleurotus ostreatus* Flour. Foods.

[146] Horita C.N., Farias-Campomanes A.M., Barbosa T.S., Esmerino E.A., da Cruz A.G., Bolini H.M.A., Meireles M.A.A., Pollonio M.A.R. (2016). The antimicrobial, antioxidant and sensory properties of garlic and its derivatives in Brazilian low-sodium frankfurters along shelf-life. Food Res. Int..

[147] Alves L.A.A.D., Lorenzo J.M., Goncalves C.A.A., dos Santos B.A., Heck R.T., Cichoski A.J., Campagnol P.B.C. (2017). Impact of lysine and liquid smoke as flavor enhancers on the quality of low-fat Bologna-type sausages with 50% replacement of NaCl by KCl. Meat Sci..

[148] Da Silva S.L., Lorenzo J.M., Machado J.M., Manfio M., Cichoski A.J., Fries L.L.M., Morgano M.A., Campagnol P.C.B. (2020). Application of arginine and histidine to improve the technological and sensory properties of low-fat and low-sodium Bologna-type sausages produced with high levels of KCl. Meat Sci..

[149] Barbieri G., Barbieri Ge., Bergamaschi M., Francheschini M., Berizi E. (2016). Reduction of NaCl in cooked ham by modification of the cooking process and addition of seaweed extract (*Palmaria palmata*). LWT Food Sci Technol..

[150] Fellendorf S., O’Sullivan M.G., Kerry J.P. (2015). Impact of varying salt and fat levels on the physicochemical properties and sensory quality of white pudding. Meat Sci..

[151] García-Lomillo J, González-SanJosé M.A., Del Pino-García R., Rivero-Pérez M.A., Muñiz-Rodríguez P. (2017). Alternative natural seasoning to improve the microbial stability of low-salt beef patties. Food Chem..

[152] De Almeida M.A., Villanueva N.D.M., Pinto J.S.D., Saldana E., Contreras-Castillo C.J. (2016). Sensory and physicochemical characteristics of low sodium salami. Sci. Agric..

[153] Pietrasik Z., Gaudette N.J. (2015). The effect of salt replacers and flavor enhancer on the processing characteristics and consumer acceptance of turkey sausages. J. Sci. Food Agric..

[154] Lobo F., Ventanas S., Morcuende D., Estevez M. (2016). Underlying chemical mechanisms of the contradictory effects of NaCl reduction on the redox-state of meat proteins in fermented sausages. LWT Food Sci. Technol..

[155] Laranjo M., Gomes A., Agulheiro-Santos A.C., Potes M.E., Cabrita M.J., Garcia R., Rocha J.M., Roseiro L.C., Fernandes M.J., Fernandes M.H. (2016). Characterisation of “Catalao” and “Salsichao” Portuguese traditional sausages with salt reduction. Meat Sci..

[156] Laranjo M., Gomes A., Agulheiro-Santos A.C., Potes M.E., Cabrita M.J., Garcia R., Rocha J.M., Roseiro L.C., Fernandes M.J., Fragueza M.J. (2017). Impact of salt reduction on biogenic amines, fatty acids, microbiota, texture and sensory profile in traditional blood dry-cured sausages. Food Chem..

[157] Flores M., Corral S., Cano-Garcia L., Salvador A., Belloch C. (2015). Yeast strains as potential aroma enhancers in dry fermented sausages. Int. J. Food Microbiol..

[158] Corral S., Salvador A., Belloch C., Flores M. (2015). Improvement the aroma of reduced fat and salt fermented sausages by *Debaromyces hansenii* inoculation. Food Control.

[159] Mora-Gallego H., Guardia M.D., Serra X., Gou P., Arnau J. (2016). Sensory characterisation and consumer acceptability of potassium chloride and sunflower oil addition in small-caliber non-acid fermented sausages with a reduced content of sodium chloride and fat. Meat Sci..

[160] Muchaamba F., Stoffers H., Blase R., Ah U.V., Tasara T. (2021). Potassium Lactate as a Strategy for Sodium Content Reduction without Compromising Salt-Associated Antimicrobial Activity in Salami. Foods.

[161] Karolenko C., Muriana P. (2020). Quantification of Process Lethality (5-Log Reduction) of *Salmonella* and Salt Concentration during Sodium Replacement in Biltong Marinade. Foods.

[162] Fougy L., Desmonts M.H., Coeuret G., Fassel C., Hamon E., Hezard B., Champomier-Verges M.C., Chaillou S. (2016). Reducing Salt in Raw Pork Sausages Increases Spoilage and Correlates with Reduced Bacterial Diversity. Appl. Environ. Microbiol..

[163] García-Díez J., Patarata L. (2017). Influence of salt level, starter culture, fermentable carbohydrates, and temperature on the behaviour of *L. monocytogenes* in sliced chouriço during storage. Acta Alim..

[164] Rodrigues F.M., Rosenthal A., Tiburski J.H., da Cruz A.G. (2016). Alternatives to reduce sodium in processed foods and the potential of high pressure technology. Food Sci. Technol..

[165] Rodrigues I., Trindade M.A., Caramit F.R., Candogan K., Pokhrel P.R., Barbosa-Canovas G.V. (2016). Effect of high pressure processing on physicochemical and microbiological properties of marinated beef with reduced sodium content. Innov. Food Sci. Emerg. Technol..

[166] Yang H.J., Han M.Y., Wang X., Han Y.Q., Wu J.Q., Xu X.L., Zhou G.H. (2015). Effect of high pressure on cooking losses and functional properties of reduced-fat and reduced-salt pork sausage emulsions. Innov. Food Sci. Emerg. Technol..

[167] Ros-Polski V., Koutchma T., Xue J., Defelice C., Balamurubam S. (2015). Effects of high hydrostatic pressure processing parameters and NaCl concentration on the physical properties, texture and quality of white chicken meat. Innov. Food Sci Emerg. Technol..

[168] Tamm A., Bolumar T., Bajovic B., Toepfl S. (2016). Salt (NaCl) reduction in cooked ham by a combined approach of high pressure treatment and the salt replacer KCl. Innov. Food Sci. Emerg. Technol..

[169] Balamurugan S., Ahmed R., Chibeu A., Gao A.L., Koutchma T., Strange P. (2016). Effect of salt types and concentrations on the high-pressure inactivation of Listeria monocytogenes in ground chicken. Int. J. Food Microbiol..

[170] Gómez-Salazar J.A., Galván-Navaro A., Lorenzo J.M., Sosa-Moráles M.E. (2021). Ultrasound effect on salt reduction in meat products: A review. Curr. Opin. Food Sci..

[171] Ojha K.S., Keenan D.F., Bright A., Kerry J.P., Tiwary B.K. (2016). Ultrasound assisted diffusion of sodium salt replacer and effect on physicochemical properties of pork meat. Int. J. Food Sci. Technol..

[172] Barretto T.L., Pollonio M.A.R., Telis-Romero J., da Silva Barretto A.C. (2018). Improving sensory acceptance and physicochemical properties by ultrasound application to restructured cooked ham with salt (NaCl) reduction. Meat Sci..

[173] Rizo A., Fuentes A., Barat J.M., Fernández-Segovia I. (2018). Development of a novel smoke-flavoured salmon product by sodium replacement using water vapour permeable bags. J. Sci. Food Agric..

[174] Vaz Leães S.Y., Basso Pinton M., de Aguiar Rosa T.C., Robalo S.S., Wagner R., de Menezes R.C., Barin S.J., Campagnol C.B.P., Cichoski A.J. (2020). Ultrasound and basic electrolyzed water: A green approach to reduce the technological defects caused by NaCl reduction in meat emulsions. Ultrason. Sonochem..

[175] Bhat Z.F., Morton J.D., Mason S.L., Bekhit A.E.A. (2020). The application of pulsed electric field as a sodium reducing strategy for meat products. Food Chem..

[176] Kim T.-K., Yong H.-I., Jung S., Kim H.-W., Choi Y.-S. (2021). Technologies for the production of meat products with a low sodium chloride content and improved quality characteristics—A review. Foods.

[177] Bonfim R.C., Machado J.D., Mathias S.P., Rosenthal A. (2015). Application of microbial transglutaminase in processed meat products with reduced content of sodium. Cienc. Rural.

[178] Martelo-Vidal M.J., Guerra-Rodriguez E., Pita-Calvo C., Vazquez M. (2016). Reduced-salt restructured European hake (*Merluccius merluccius*) obtained using microbial transglutaminase. Innov. Food Sci. Emerg. Technol..

